# Final OS analyses from the TOURMALINE- MM3 and -MM4 RCTs of ixazomib maintenance in newly diagnosed multiple myeloma

**DOI:** 10.1038/s41408-025-01411-9

**Published:** 2025-12-04

**Authors:** Meletios A. Dimopoulos, Sagar Lonial, Wee-Joo Chng, Shinsuke Iida, María-Victoria Mateos, Gareth J. Morgan, Cong Li, Catriona Byrne, Kaveri Suryanarayan, Richard Labotka, S. Vincent Rajkumar

**Affiliations:** 1 Department of Clinical Therapeutics, School of Medicine, National and Kapodistrian University of Athens, Athens, Greece & Department of Medicine, Korea University, Seoul, South Korea; 2 Department of Hematology and Medical Oncology, Winship Cancer Institute, Emory University Medical School, Emory University, Atlanta, GA USA; 3 Department of Hematology-Oncology, National University Cancer Institute Singapore, Singapore, Singapore; 4 Department of Hematology and Oncology, Nagoya City University Graduate School of Medical Sciences, Nagoya, Japan; 5 University Hospital of Salamanca, CIC, IBMCC, Salamanca, Spain; 6 Perlmutter Cancer Center, NYU Langone Health, New York, NY USA; 7 Takeda Development Center Americas, Inc. (TDCA), Cambridge, MA USA; 8 Division of Hematology, Department of Internal Medicine, Mayo Clinic, Rochester, MN USA

**Keywords:** Chemotherapy, Myeloma

## Abstract

TOURMALINE-MM3 (NCT02181413) and -MM4 (NCT02312258) were phase 3 studies of fixed-duration, single-agent ixazomib maintenance in post-transplant (TOURMALINE-MM3)/transplant-ineligible (TOURMALINE-MM4) patients with newly diagnosed multiple myeloma (NDMM) that demonstrated improved median progression-free survival (PFS) for ixazomib vs placebo. We present the final overall survival (OS) analyses for each study separately. In both studies, eligible patients were randomized 3:2 to receive ixazomib maintenance (3 mg [cycles 1–4], 4 mg [from cycle 5 if tolerated]) or matching placebo for ≤26 cycles, or until progressive disease/unacceptable toxicity. At median follow-up of approximately 8 years (TOURMALINE-MM3) and 5 years (TOURMALINE-MM4), median OS was not reached in either arm in MM3 (hazard ratio [HR], 1.025; 95% confidence interval [CI], 0.789–1.332; *p* = 0.850), and was 64.8 (ixazomib) vs 69.5 (placebo) months in MM4 (HR, 1.090; 95% CI, 0.861–1.381; *p* = 0.473). No new safety signals were identified in either study; incidence of new primary malignancies was low. Despite meeting their primary endpoints (PFS), neither final OS analysis of TOURMALINE-MM3/-MM4 showed statistically significant differences between fixed-duration ixazomib maintenance and placebo in patients with NDMM. The growing number of available, highly effective salvage treatments with novel mechanisms of action make demonstrating an OS advantage in front-line myeloma studies increasingly challenging.

## Introduction

The increasing number of treatment options available for patients with multiple myeloma (MM) has been associated with improvement in progression-free survival (PFS) and overall survival (OS), but effective and tolerable therapies are still required, particularly for hard-to-treat groups, such as elderly patients, and those with high-risk cytogenetic abnormalities [1, 2]. Considering the guideline-recommended use of lenalidomide in earlier lines of therapy, and the approval of lenalidomide as post-autologous stem cell transplantation (ASCT) maintenance in MM, the population of lenalidomide-exposed and -refractory patients is increasing [3, 4]. Proteasome inhibitors (PIs), such as bortezomib, provide an alternative to lenalidomide due to their different mechanism of action [5]; however, prolonged use of parenteral PIs as maintenance therapy may be limited due to treatment burden [6]. Ixazomib is an oral PI with once-weekly dosing that is approved in Europe and the United States in combination with lenalidomide and dexamethasone for the treatment of patients with relapsed/refractory MM who have received at least one prior therapy [7, 8]. Based on the TOURMALINE-MM3 and TOURMALINE-MM4 studies [2, 9], ixazomib has also been approved as maintenance therapy in Japan (March 2020/May 2021), South Korea (March/September 2021), Thailand (July 2022), and Philippines (April 2023) for both post-transplant and transplant-ineligible newly diagnosed MM (NDMM) patients, and in Brazil (August 2022) in the transplant-ineligible maintenance setting.

TOURMALINE-MM3 and TOURMALINE-MM4 (hereafter referred to as MM3 and MM4, respectively) were two global, randomized, double-blinded, placebo-controlled phase 3 studies of fixed-duration single-agent ixazomib maintenance therapy in patients with NDMM following primary therapy [2, 9]. MM3 (NCT02181413) included patients with at least a partial response (PR) following ASCT [9], while MM4 (NCT02312258) enrolled patients not undergoing ASCT with at least a PR following 6–12 months’ standard induction therapy [2]. Both studies demonstrated a statistically significant and clinically meaningful improvement in the primary endpoint of PFS with ixazomib vs placebo (MM3: improvement of 5.2 months, median 26.5 vs 21.3 months [hazard ratio (HR), 0.72; 95% confidence interval (CI), 0.58–0.89; *p* = 0.002] [9]; MM4: improvement of 8.0 months, median 17.4 vs 9.4 months [HR, 0.66; 95% CI, 0.54–0.80; *p* < 0.001]) [2].

However, interim analyses of OS for MM3 and MM4 did not demonstrate a statistically significant difference between ixazomib and placebo [10]. After 64 months of follow-up in MM3, OS did not differ between treatment arms (5-year Kaplan–Meier estimates of OS were 74% for ixazomib vs 73% for placebo; median OS was not reached (NR) in either arm [HR, 1.008; 95% CI, 0.744–1.367; *p* = 0.958]) [10]. Sensitivity analyses suggested a trend favoring ixazomib when adjusting for confounding factors, by Kaplan–Meier estimates with inverse-probability-of-censoring weighted (IPCW) averages (HR, 0.623; 95% CI, 0.190–2.045; *p* = 0.436) and marginal structural model (MSM) analysis (HR, 0.725; 95% CI, 0.184–2.851; *p* = 0.645) [10]. For MM4, after 36 months of follow-up, the 5-year Kaplan–Meier estimates were 55% for ixazomib vs 56% for placebo and the median OS was NR in either arm (HR, 1.136; 95% CI, 0.853–1.514; *p* = 0.382); sensitivity analyses were consistent with the primary analysis [10].

While OS is a good indicator of efficacy and has historically represented a key endpoint in myeloma randomized controlled trials (RCTs) [11], the evolving treatment landscape and a growing number of highly effective salvage therapies for MM [12–14] means that subsequent therapies cannot be accounted for during RCT design, and therefore OS may no longer be a tenable endpoint in this setting.

Here we report the final OS analysis for each of these two studies.

## Methods

### Patients and treatment

Study designs and methodologies have been reported previously [2, 9]. Briefly, the MM3 study included patients who had received a PI and/or immunomodulatory drug induction therapy followed by a single ASCT within 12 months of diagnosis (randomization ≤15 days after screening and ≤115 days after ASCT; stratification by induction regimen, preinduction International Staging System (ISS) disease stage, and response after ASCT). Patients in MM4 were transplant-ineligible/unwilling/unable to undergo ASCT and had 6–12 months’ standard-of-care induction (randomization ≤60 days after last dose of induction; stratification by induction regimen, ISS disease stage, age at randomization, and response before randomization).

In both studies, eligible patients were randomized 3:2 to receive maintenance therapy with fixed-duration single-agent ixazomib 3 mg or matching placebo (days 1, 8, and 15 of 28-day cycles) for up to 24 months (26 cycles) or until progressive disease or unacceptable toxicity, whichever occurred first. The dose was increased to 4 mg with the same dosing schedule after cycle 5 if ixazomib was well tolerated during cycles 1–4. See the Supplementary Methods for further details.

The studies were conducted in accordance with the principles of the Declaration of Helsinki and the International Conference on Harmonization Good Clinical Practice guidelines, and applicable local regulatory requirement(s). Study documents were reviewed and approved by local independent ethics committees (IECs) or institutional reviews boards (IRBs) (please refer to the Supplementary Materials for the full list or IECs and IRBs for each study). All patients provided informed consent.

### Outcomes and assessments

In both studies, the primary endpoint was PFS, assessed by blinded independent review, and has been previously described [2, 9]. OS was a prespecified key secondary endpoint. Other secondary endpoints included: OS and PFS in high-risk cytogenetic populations; PFS on next-line treatment (PFS2); time to next treatment (TTNT); incidence of new primary malignancies (NPM); safety; and health-related quality of life (HRQoL), as measured by the global health domain of the European Organisation for the Research and Treatment of Cancer (EORTC) Quality of Life Questionnaire Core-30 (QLQ-C30) [9]. A pre-specified sensitivity analysis of OS was also conducted to adjust for the potential effect of subsequent therapy. Exploratory ad hoc analyses included assessments of subsequent anti-myeloma therapies and OS in patients whose next line of therapy included a PI vs not.

### Statistical analysis

Detailed randomization stratification and statistical analyses are described in the Supplementary Methods. PFS (defined as the time from randomization to the first documentation of disease progression or death due to any cause, whichever occurs first) and OS (defined as the time from randomization to death) for both MM3 and MM4 studies were evaluated by closed sequential-testing procedures and based on the intention-to-treat (ITT) populations (defined as all patients who were randomized and have post-randomization data). OS was assessed using an O’Brien-Fleming alpha-spending function (Lan-Demets method) following demonstration of a significant PFS difference [9]. OS distributions were analyzed using the Kaplan–Meier methodology, with stratified log rank tests and Cox models for between-group comparisons. An unadjusted stratified Cox model was used to estimate the HR and its 95% CIs for the treatment effect using the stratification factors. OS was further evaluated within the following prespecified patient subgroups: age (<60 vs ≥60 and <75 years); preinduction ISS (I vs II vs III); response at study entry (complete response vs very good PR or less); cytogenic risk (high risk vs standard risk); induction regimen (PI-exposed vs PI-naïve). Exploratory subgroup analyses by minimal residual disease (MRD) status at study entry and frailty status were also conducted. To adjust for the potential effects of subsequent therapies after patients discontinued study treatment, MSM [15] and IPCW [16] methods were used. See the Supplementary Methods for further details.

## Results

### Patient disposition

#### MM3

**Fig. 1:**
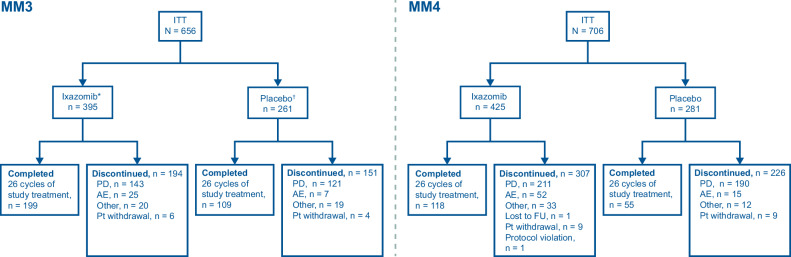
Disposition of patients during the TOURMALINE-MM3 and TOURMALINE-MM4 studies. *Two patients in the ixazomib arm did not receive the allocated treatment (one patient withdrew, and the other one did not meet the inclusion criteria due to a low platelet count) [9]. ^†^One patient in the placebo arm did not receive the allocated treatment due to patient withdrawal [9]. AE adverse event, FU follow-up, ITT intent-to-treat, PD progressive disease, Pt patient.

At the time of data cutoff (September 8, 2023), 194/395 (49%) patients in the ixazomib arm vs 151/261 (58%) patients in the placebo arm had discontinued study treatment prior to the completion of 24 months of therapy (or 26 cycles) (Fig. 1). In the ixazomib vs placebo arms, 74% vs 80% of discontinuations were due to PD and 13% vs 5% were due to adverse events.

#### MM4

At the time of data cutoff (October 29, 2022), 307/425 (72%) patients in the ixazomib arm vs 226/281 (80%) patients in the placebo arm had discontinued study treatment prior to the completion of 24 months of therapy. In the ixazomib vs placebo arms, 69% vs 84% of discontinuations were due to PD and 17% vs 7% were due to adverse events (Fig. 1).

### Patient demographics and baseline characteristics

#### MM3

Patient demographics and baseline characteristics have been published previously and were generally balanced between treatment arms (Supplementary Table S1) [9]. The median age was 58 and 60 years in the ixazomib and placebo arms, respectively; 15% and 21% of patients had high-risk cytogenetic abnormalities (del[17p] and/or t[4;14] and/or t[14;16]). Of 585/656 patients tested for MRD, 225/357 (63%) vs 139/228 (61%) were MRD-positive in the ixazomib vs placebo arms, while 117/357 (33%) vs 75/228 (33%) were MRD-negative.

#### MM4

Patient demographics and baseline characteristics have been published previously and were generally balanced between treatment arms (Supplementary Table S2) [2]. In the ixazomib and placebo arms, the median age was 72 and 73 years; 24% of patients in both arms were classified as frail [17–19], and high-risk cytogenetic abnormalities were present in 17% of patients in both arms. Most patients (87%) were deemed transplant-ineligible because of their age. Of 364/706 patients tested for MRD, 169/213 (79%) vs 125/151 (83%) were MRD-positive in the ixazomib vs placebo arms, and 44/213 (21%) vs 26/151 (17%) were MRD-negative.

### Final OS analysis

#### MM3

**Fig. 2:**
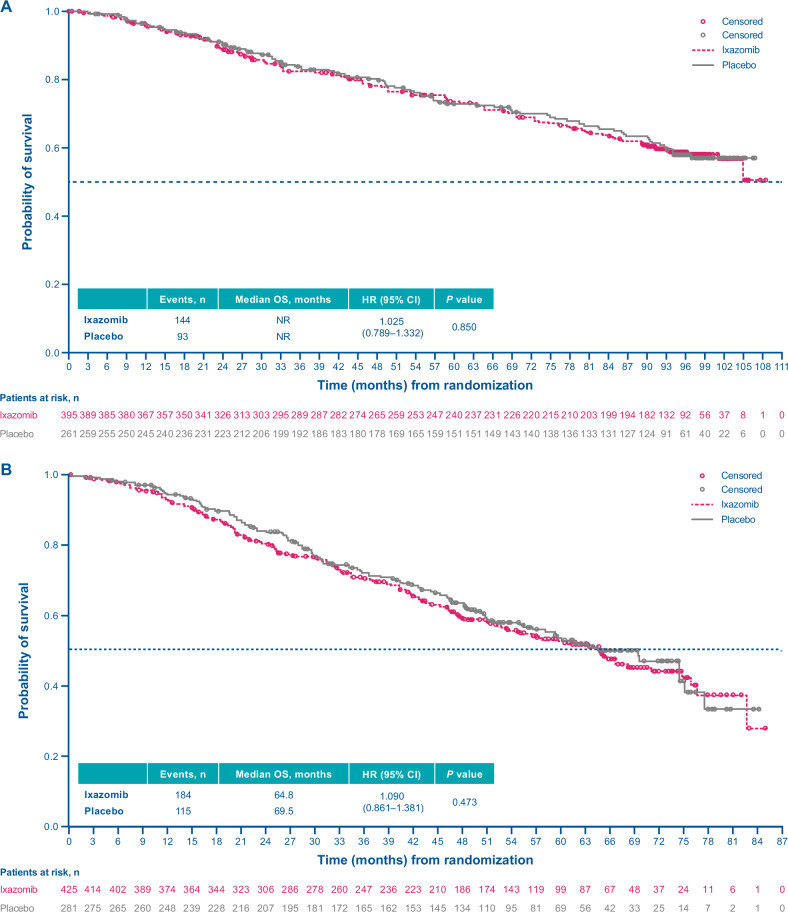
Kaplan–Meier analysis of overall survival (ITT population). **A** TOURMALINE-MM3 study. **B** TOURMALINE-MM4 study. CI confidence interval, HR hazard ratio, NR not reached, OS overall survival.

**Fig. 3:**
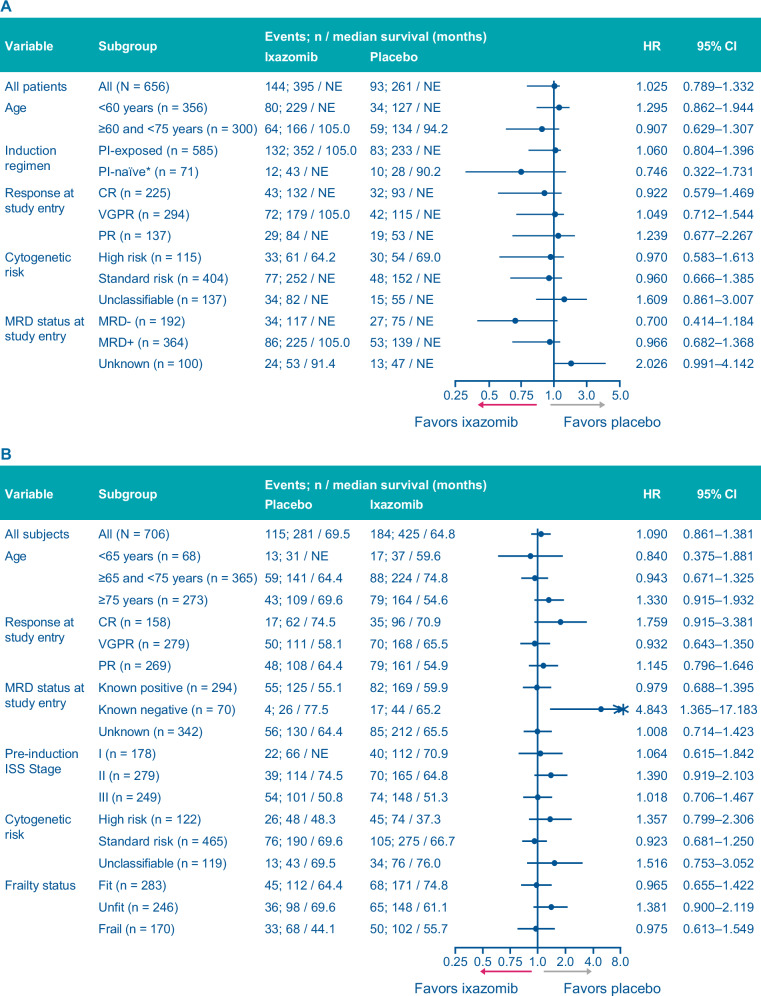
Overall survival in patient subgroups (ITT population). **A** TOURMALINE-MM3 study. **B** TOURMALINE-MM4 study. CI confidence interval, CR complete response, HR hazard ratio, ISS International Staging System, ITT intention-to-treat, MRD minimal residual disease, NE not estimable, PI proteasome inhibitor, PR partial response, VGPR very good partial response.

At a median follow-up of 94.4 months in the ixazomib arm vs 94.5 months in the placebo arm, 144 (36%) vs 93 (36%) patients had died, and the median OS was NR in either arm (HR, 1.025; 95% CI, 0.789–1.332; *p* = 0.850) (Fig. 2A). There were no statistically significant differences in OS with ixazomib vs placebo among pre-specified subgroups of patients (Fig. 3A). However, there was a trend in favor of ixazomib among patients who were PI-naïve (HR, 0.746; 95% CI, 0.322–1.731) or MRD-negative at study entry (HR, 0.700; 95% CI, 0.414–1.184) (Fig. 3A).

#### MM4

At a median follow-up of 57.2 months in the ixazomib arm vs 57.6 months in the placebo arm, 184 patients (43%) vs 115 patients (41%) had died, and the median OS was 64.8 months in the ixazomib arm vs 69.5 months in the placebo arm (HR, 1.090; 95% CI, 0.861–1.381; *p* = 0.473) (Fig. 2B). There were no statistically significant differences in OS with ixazomib vs placebo among pre-specified subgroups of patients (Fig. 3B).

### Subsequent therapy

#### MM3

**Fig. 4:**
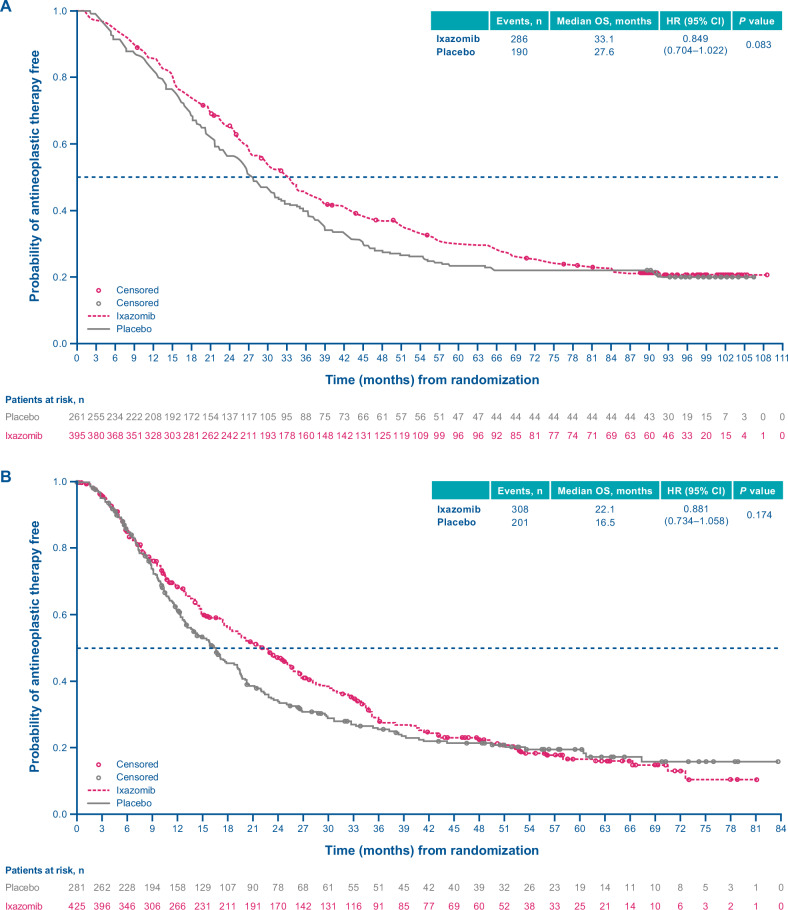
Kaplan–Meier analysis of time to next treatment (ITT population). **A** TOURMALINE-MM3 study. **B** TOURMALINE-MM4 study. CI confidence interval, HR hazard ratio, ITT intention-to-treat, OS overall survival.

**Fig. 5:**
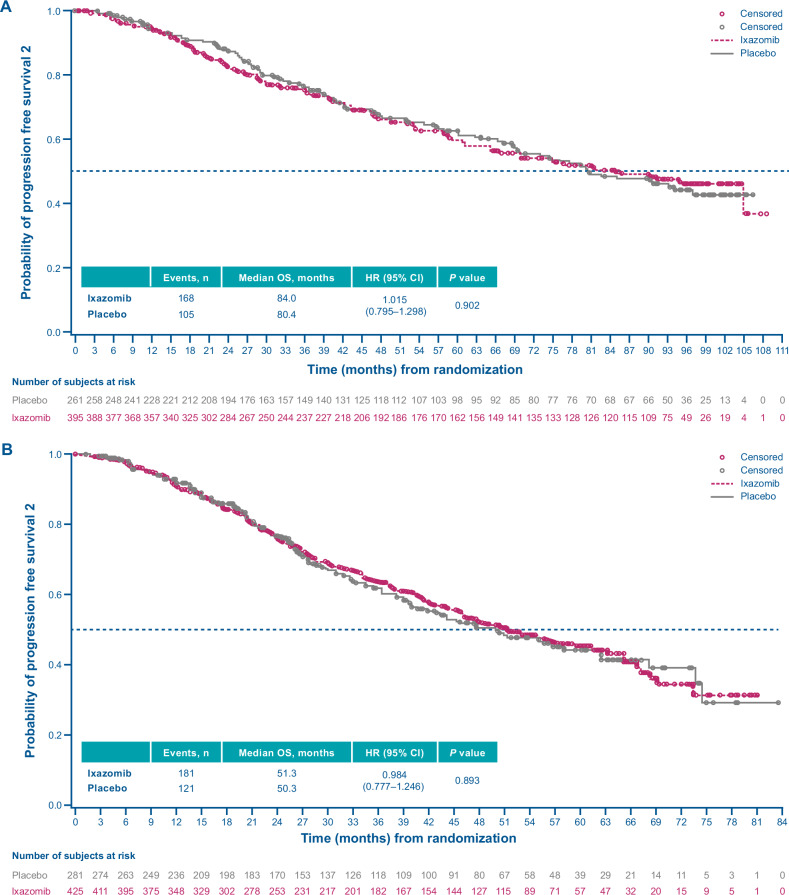
Progression-free survival in next-line therapy (ITT population). **A** TOURMALINE-MM3 study. **B** TOURMALINE MM4 study. CI confidence interval, HR hazard ratio, ITT intention-to-treat, OS overall survival.

The median TTNT (ITT population) was 33.1 vs 27.6 months with ixazomib vs placebo (HR, 0.849; 95% CI, 0.704–1.022; *p* = 0.083) (Fig. 4); median PFS2 was 84.0 vs 80.4 months, respectively (HR, 1.015; 95% CI, 0.795–1.298; *p* = 0.902) (Fig. 5).

In total, 73% of patients (safety population) in each treatment arm received ≥1 subsequent therapy (Table 1). Subsequent therapies with ≥5% rate difference between arms (ixazomib vs placebo) included PIs (46.2% vs 54.4%) and anti-CD38 antibodies (35.0% vs 28.6%).

**Table 1:** Subsequent therapies (safety population).

	TOURMALINE-MM3		TOURMALINE-MM4	
	Ixazomib (n = 394)	Placebo (n = 259)	Ixazomib (n = 426)	Placebo (n = 276)
≥1 subsequent anti-cancer therapy, n (%)	288 (73.1)	190 (73.4)	308 (72.3)	198 (71.7)
Corticosteroids	263 (66.8)	169 (65.3)	286 (67.1)	184 (66.7)
IMiDs	255 (64.7)	161 (62.2)	257 (60.3)	168 (60.9)
PIs	182 (46.2)	141 (54.4)	143 (33.6)	99 (35.9)
Anti-CD38 antibodies	138 (35.0)	74 (28.6)	109 (25.6)	73 (26.4)
Alkylating agents	105 (26.6)	71 (27.4)	99 (23.2)	58 (21.0)

*IMiD* immunomodulatory drug, *PI* proteasome inhibitor.

In sensitivity analyses adjusting for differences in subsequent therapies received, there was no difference in OS between the ixazomib arm and the placebo arm using either method (MSM: HR, 0.925; 95% CI, 0.261–3.279; *p* = 0.904; IPCW: HR, 0.784, 95% CI, 0.252–2.446; *p* = 0.676).

**Fig. 6:**
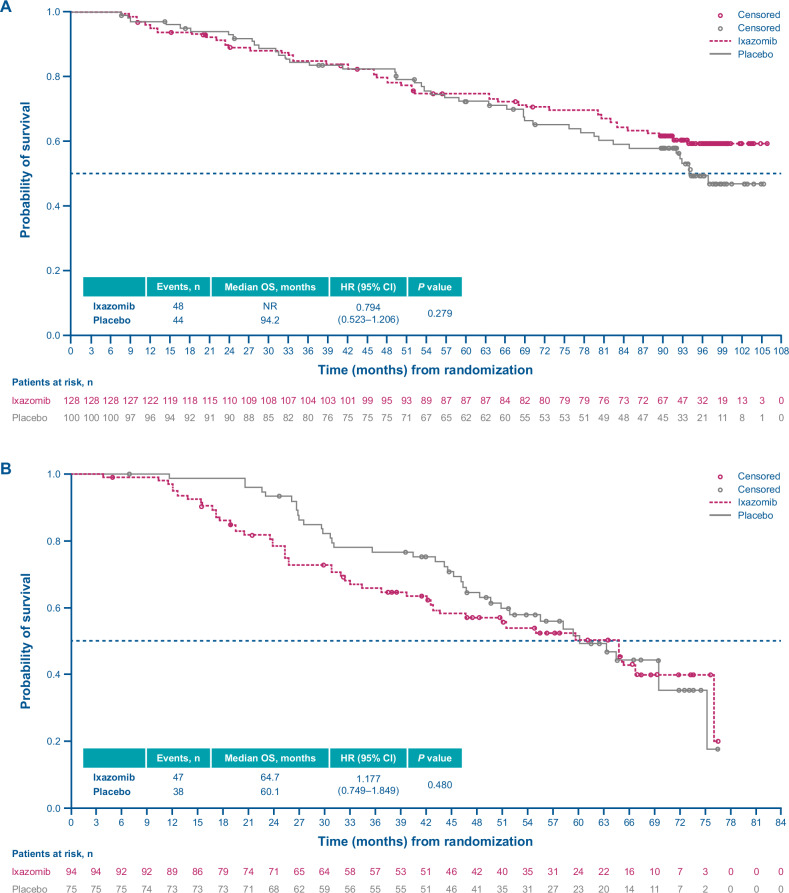
Kaplan–Meier analysis of overall survival in patients who received a proteasome inhibitor as next-line therapy. **A** and TOURMALINE-MM3 study. **B** TOURMALINE-MM4 study. CI confidence interval, HR hazard ratio, OS overall survival.

**Fig. 7:**
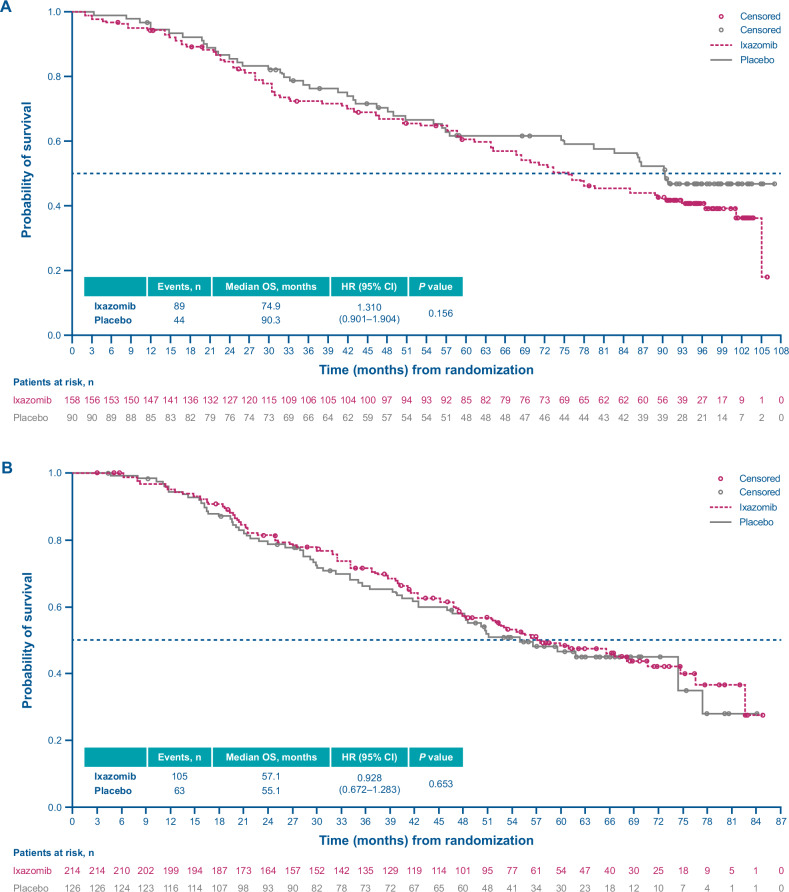
Kaplan–Meier analysis of overall survival in patients whose next-line therapy did not include a proteasome inhibitor. **A** TOURMALINE-MM3 study. **B** TOURMALINE-MM4 study. CI confidence interval, HR hazard ratio, OS overall survival.

In exploratory ad hoc analyses, among patients who received a PI as their next line of therapy, median OS was NR in the ixazomib arm vs 94.2 months in the placebo arm (HR, 0.794; 95% CI, 0.523–1.206) (Fig. 6A); in patients whose next line of therapy did not include a PI, the median OS was 74.9 vs 90.3 months for ixazomib vs placebo (HR, 1.310; 95% CI, 0.901–1.904) (Fig. 7A).

#### MM4

Median TTNT (ITT population) was 22.1 vs 16.5 months with ixazomib vs placebo (HR, 0.881; 95% CI, 0.734–1.058; *p* = 0.174) (Fig. 4); median PFS2 was 51.3 vs 50.3 months, respectively (HR, 0.984; 95% CI, 0.777–1.246; *p* = 0.893) (Fig. 5).

In total, 72% of patients (safety population) in each treatment arm received ≥1 subsequent therapy; between-arm rates of subsequent therapies were all within 5% of each other (Table 1).

In sensitivity analyses adjusting for differences in subsequent therapies received, there was no difference in OS between the ixazomib arm and the placebo arm using either method (MSM: HR, 1.190; 95% CI, 0.897–1.577; *p* = 0.227; IPCW: HR, 1.160, 95% CI, 0.579–2.323; *p* = 0.676).

In exploratory ad hoc analyses, median OS was 64.7 vs 60.1 months for ixazomib vs placebo (HR, 1.177; 95% CI, 0.749–1.849) in patients whose next line of therapy included a PI (Fig. 6B), and 57.1 vs 55.1 months (HR, 0.928; 95% CI, 0.672–1.283) among patients whose next line of therapy did not include a PI (Fig. 7B).

### Safety summary

#### MM3

By the time of the primary analysis, all patients had completed the fixed-duration maintenance treatment and thus no new safety data were collected beyond NPMs and those already published [9]. The incidence of NPMs in the ixazomib vs placebo arms was 7% vs 8%; incidences of hematological NPMs were 2% vs 3%, respectively (Supplementary Table S4).

#### MM4

A small number of patients stayed on treatment after the primary analysis, and so we present additional safety data for the study in Supplementary Table S3; no new safety signals with ixazomib were identified. While on-study deaths occurred in 11 patients in the ixazomib arm [sepsis (n = 2), septic shock (n = 4), pneumonia viral, acute pulmonary edema, acute kidney injury, plasma cell myeloma, cardiac arrest all (n = 1)], only one was considered to be associated with the study treatment; among the six patients who died in the placebo arm [respiratory failure, septic shock, sudden death, myocardial infarction (all n = 1) and plasma cell myeloma (n = 2)], none were treatment-related. The incidence of NPMs in each treatment arm was 8%, with <1% incidence of hematological NPMs in either arm (Supplementary Table S4).

#### HRQoL

In both studies, HRQoL was maintained during the protocol-defined treatment period in both arms. Scores on the EORTC QLQ-C30 global health status/QoL scale were stable and generally similar with fixed-duration ixazomib maintenance and with placebo throughout the treatment period and at end of treatment (Supplementary Fig. S1).

## Discussion

Despite meeting their primary endpoint of improved PFS [2, 9], the final OS analyses for the TOURMALINE-MM3 and -MM4 studies demonstrated no statistically significant differences between fixed-duration ixazomib maintenance and placebo. After approximately 94 months of follow-up in MM3 and 57 months of follow-up in MM4, OS did not differ between treatment arms. Additionally, there were no statistically significant differences in OS observed between ixazomib and placebo among patient subgroups in either study, including high-risk patients. Of note, 32% and 26% of patients in MM3 and MM4, respectively, received subsequent anti-CD38 therapy following relapse, which, in combination with immunomodulatory drugs/PIs and dexamethasone, have been shown to improve PFS and OS outcomes among patients with relapsed/refractoryMM [20] and may have confounded results. In pre-specified sensitivity analyses adjusting for differences in subsequent therapies received, no differences in OS between ixazomib and placebo were observed in either study; however, based upon an exploratory analysis, OS was found to be confounded by subsequent therapies in both studies, limiting any further clinical interpretation of these results. This highlights that although OS is a good indicator of efficacy, and a key endpoint in myeloma trials, RCTs do not currently control for subsequent therapies [21, 22], and thus OS may no longer be a viable endpoint in this setting.

Lenalidomide maintenance therapy, regarded as the cornerstone treatment in patients with NDMM after ASCT [23], often follows prior exposure with lenalidomide induction and is administered until disease progression; whereas the fixed-duration ixazomib maintenance in MM3 and MM4 was administered for a maximum duration of 2 years following standard-of-care induction (not including ixazomib) and/or ASCT. Therefore, it is possible that optimal efficacy results were not obtained in the MM3 and MM4 studies due to the limited course of treatment. In a network meta-analysis (2018), lenalidomide maintenance (ranging between 2 and 3 years to disease progression) was considered the best treatment option for patients with NDMM, based on PFS and OS data [24]. However, a real-world study conducted by the Danish Myeloma Study Group found that OS in patients receiving lenalidomide maintenance was similar to a historical cohort not receiving lenalidomide maintenance (unadjusted HR, 1.22; 95% CI, 0.78–1.89). The majority of early discontinuations in this study were due to toxicity [25].

Despite almost 8 years’ follow-up in MM3 in patients who had undergone prior ASCT, the median duration of OS was NR for both ixazomib and placebo. Although the evolution of myeloma treatment in the last decade makes comparison between studies carried out in different eras very challenging, the OS seen in the placebo arm in MM3 is equivalent to, and in some cases exceeds, the OS reported in the active treatment arm of lenalidomide maintenance studies. For example, in the ATLAS study, median OS with lenalidomide maintenance (given until disease progression or unacceptable toxicity) was 61.8 months (approximately 5.2 years) [23]. In CALGB 100104, median OS with lenalidomide maintenance (given until disease progression) was 113.8 months (approximately 9.5 years) [26]. In the FORTE study [27], OS with lenalidomide maintenance (given until progression or intolerance) was NR after a median follow-up of 37.3 months (approximately 3.1 years), with similar results reported in two retrospective studies [28, 29].

In the DETERMINATION study, despite a 21-month improvement in PFS with lenalidomide, bortezomib, and dexamethasone (RVd) followed by ASCT vs RVd alone in patients with NDMM, the study did not demonstrate an OS improvement, with the impact of novel subsequent therapies contributing to the distinct difference between PFS and OS outcomes [22]. Similarly, in MM4, although there was a near doubling of PFS with fixed-duration ixazomib vs placebo (9.4 months; HR, 0.659; *p* < 0.001) [2], this did not translate into OS benefit. These data are consistent with most maintenance studies regardless of transplant eligibility [30–32].

Due to the double-blind nature of the MM3 and MM4 studies, and following disease progression, more than one third of patients (46% and 34%, respectively) who received ixazomib received another PI to which in some instances they were already refractory to. In MM4 (transplant-ineligible), for patients who received a PI as next line of therapy, a slight numerical OS benefit with placebo was observed (HR, 1.177), and for patients whose next line of therapy did not include a PI, a slight OS benefit with ixazomib was observed (HR, 0.928). We can hypothesize that most of the ixazomib-treated patients were still receiving ixazomib when disease progression occurred due to the 24-month treatment period (median PFS, 17.4 months), and so these patients developed refractoriness to PIs during this treatment period. However, the opposite was seen in the MM3 study (following ASCT); for patients who received a PI as next line of therapy, OS benefit with ixazomib was observed (HR, 0.794), with most patients who experienced disease progression in the ixazomib treatment group having done so after completing study treatment (median PFS, 26.5 months). This suggests that more patients remained sensitive to PIs as they had not been treated to disease progression, providing an argument for longer maintenance treatment with ixazomib in this setting. Although some PI sensitivity may be retained when a different PI is used, since subsequent therapies were not controlled in these two studies, this hypothesis could not be tested. In contrast, among patients in MM3 whose next line of therapy did not include a PI, a numerical OS benefit with placebo was observed (HR, 1.310); however, it is important to note that the Kaplan–Meier curves for OS only separate around 60 months, or approximately 3 years after completion of maintenance (Fig. 4A).

Despite the lack of an OS advantage demonstrated in both MM3 and MM4 studies, ixazomib maintenance, given for a fixed duration of 2 years, provided a statistically significant and clinically meaningful PFS benefit for patients with NDMM [2, 9]. Fixed-duration ixazomib maintenance was well tolerated, with no new safety signals identified, a low incidence of NPMs (7% in MM3 and 8% in MM4) similar to those in lenalidomide maintenance studies (ranging from 2 to 8%) [23, 26, 27, 29], and unaffected HRQoL compared with placebo. More than half of patients in MM3 and approximately a third of patients in MM4 completed 26 cycles or 2 years of ixazomib maintenance, with a very small proportion of patients discontinuing treatment due to toxicities (13% in MM3 and 17% in MM4). The slightly higher rate of discontinuation in MM4 is possibly stemming from an older patient population who were deemed transplant-ineligible. This compares favorably with lenalidomide studies, such as CALGB 100104, in which the majority of patients did not complete maintenance treatment [26]. Therefore, ixazomib can be positioned as an attractive alternative fixed-duration maintenance treatment for patients who are lenalidomide-refractory or unable to receive it. Furthermore, ixazomib might provide PFS benefit for patients with high-risk cytogenetics, as demonstrated in a pooled analysis of TOURMALINE-MM1/-MM2/-MM3/-MM4 [33], either alone or as part of a combination strategy. Exploring longer duration treatment with ixazomib may also be warranted, particularly considering the delayed separation of the OS curves observed in MM3. Based on the results of the Myeloma XI study of lenalidomide maintenance in patients with NDMM (which treated until disease progression) [31], there is agreement that lenalidomide maintenance therapy should be used for at least three years; therefore, it is possible that three years of ixazomib treatment may be required for optimal results. Nevertheless, we acknowledge that additional maintenance options, such as daratumumab, are likely to become available in the near future, following the results of the CASSIOPEIA study, which demonstrated significant improvements in PFS for transplant-eligible patients with NDMM who received daratumumab maintenance following daratumumab, bortezomib, thalidomide, and dexamethasone induction and consolidation regimen versus patients who did not receive daratumumab maintenance [34]. Emerging data also support the use of dual-agent maintenance therapies over standard monotherapy maintenance, especially for select subgroups of patients [35]. Whilst it is possible that ixazomib in combination with other agents may be an option for patients with MM in the maintenance setting, due to the previously demonstrated efficacy and tolerability of ixazomib in combination regimens in NDMM [36–38], the efficacy and safety of these combinations in a maintenance setting would need to be further assessed. Addition of ixazomib to lenalidomide maintenance in post-transplant patients with NDMM demonstrated promising PFS, and no new safety signals were observed, indicating that addition of ixazomib may provide additional benefit versus lenalidomide alone for post-transplant patients in the maintenance setting [39]. A phase 2 trial assessing the efficacy and tolerability of ixazomib in combination with daratumumab and dexamethasone (for up to 9 induction cycles), followed by maintenance treatment with ixazomib and daratumumab (for a maximum of 2 years), among frail patients with NDMM reported high response rates and improved quality of life [40]; however, premature treatment discontinuation due to toxicity/early mortality was a concern for this particular subgroup of patients, which remains an important consideration in the maintenance setting.

A key limitation of these studies is their placebo-controlled designs. Due to the timing of the study initiation, which was prior to the approval of lenalidomide as post-ASCT maintenance therapy, it does not provide a direct comparison vs the only agent approved in this setting. Further to this, patients with minimal response or stable disease after induction were not eligible for the maintenance phase, which may limit the generalizability of the findings.

In conclusion, the prolonged PFS and tolerable safety profile of ixazomib reported in MM3 and MM4, associated with the convenience of an oral therapy, makes ixazomib an alternative and viable treatment option for patients with NDMM (regardless of transplant status) who are refractory to, or unable to receive, other common maintenance options such as lenalidomide.

## Supplementary information

Supplementary material

TOURMALINE-MM3 IRB and IEC Information

TOURMALINE-MM4 IRB and IEC Information

## Data Availability

The datasets, including the redacted study protocols, redacted statistical analysis plans, and individual participant data supporting the results of the completed study will be made available after the publication of the final study results within 3 months from initial request to researchers who provide a methodologically sound proposal. The data will be provided after its de-identification, in compliance with applicable privacy laws, data protection, and requirements for consent and anonymization.
