# Supplementary material for: Final OS analyses from the TOURMALINE- MM3 and -MM4 RCTs of ixazomib maintenance in newly diagnosed multiple myeloma
Source: Blood Cancer J. 2025 Dec 4;16(1):15. doi: 10.1038/s41408-025-01411-9 (PMC12808303; doi:10.1038/s41408-025-01411-9)
Supplement: Supplementary file 1 — Supplementary material [file 41408_2025_1411_MOESM1_ESM.docx]

**Final OS analyses from the TOURMALINE- MM3 and -MM4 RCTs of ixazomib maintenance in newly diagnosed multiple myeloma**

Meletios A. Dimopoulos MD, Sagar Lonial MD, Wee-Joo Chng PhD, Shinsuke Iida MD, PhD, María-Victoria Mateos MD, PhD, Gareth J. Morgan MD, PhD, Cong Li PhD, Catriona Byrne RN, Kaveri Suryanarayan MD, Richard Labotka MD, MS, and S. Vincent Rajkumar MD

# SUPPLEMENTARY METHODS

## Patients and treatment

Eligible patients were adults with symptomatic multiple myeloma (International Myeloma Working Group criteria) [1] who had achieved ≥partial response (PR) before maintenance randomization and had an Eastern Cooperative Oncology Group performance status of 0–2.

**Statistical analysis**

Patients in both MM3 and MM4 studies were randomized 3:2 to receive ixazomib or matching placebo capsule. Randomization for MM3 was stratified by induction therapy (PI without an immunomodulatory drug [IMiD] vs IMiD without a PI vs PI and IMiD), pre-induction International Staging System (ISS; stage I vs stage II or III) and response after transplantation, defined as the response [complete response (CR) or very good partial response (VGPR) vs PR] to induction/autologous stem cell transplant (ASCT) measure during screening. For MM4, there were four stratification factors: initial therapy (PI-containing or not), ISS stage before initial therapy (stage I or II vs stage III), age at time of randomization (<75 vs ≥75 years), and best response to initial therapy, as measured during screening (CR or VGPR vs PR). The randomization scheme was generated by an independent statistician at the study sponsor who was not a member of the study team for both trials.

The order of the closed sequential-testing procedures for MM3 and MM4 was:

1. At the first interim analysis for progression-free survival (PFS; 25 months for MM3 and 10 months for MM4)
2. At the interim analyses and final analysis for overall survival (OS, once significance has been demonstrated for PFS)

Treatment comparison was tested at a two-side alpha level of 0.05 for PFS, while the significance level for OS was determined by the O’Brien-Fleming alpha spending function (the Lan-DeMets method).

Subgroup analyses for PFS and OS were performed based on selected key baseline demographics and disease characteristics such as gender, race, and age.

As standard methods for OS analysis do not adjust for time-dependent confounders and may produce biased estimates of treatment effect if patients receive subsequent therapies, a pre-specified sensitivity analysis was conducted using marginal structural models (MSM [15]) and inverse probability of censoring weighted (IPCW [16]) approaches; covariates affecting disease progression, post-progression treatment, and OS endpoint were used. Potential time-fixed covariates at study entries were region (APAC, EMEA, other), age (<60, ≥ 60 and <75), race (white, non-white), Eastern Cooperative Oncology Group performance score (0 or 1, 2), induction therapy (PI only, IMiD only, PI and IMiD), response at study entry (CR or VGPR, PR), percentage of plasma cells (≤ 30, > 30, missing), presence of extramedullary plasmacytomas (yes, no), presence of lytic bone lesions (yes, no), hemoglobin, platelets, creatinine clearance, albumin, and corrected calcium. Potential time-fixed covariates at initial diagnosis were type of myeloma (IgA, other), ISS (I, II or III), RISS (I or II, III), cytogenetic abnormalities (high risk, others), LDH, β2 microglobulin. Time-varying covariates included duration of exposure, disease progression status at each study visit, hemoglobin value at each study visit and progression/relapse visit, platelets value at each study visit and progression/relapse, M-protein value at each study visit and progression/relapse, type of subsequent therapy with proteasome inhibitor, types of subsequent therapy with IMIDs. The final criteria for selected covariates was a p-value of less than or equal to 0.1 in the multivariate logistic regression models for weight calculations. If there were more than 5% missing in the covariate, then this covariate was dropped from the weighting calculation and final OS model. For both MSM and IPCW analyses, logistic regression models on repeated measurements were used to approximate the Cox models in the weight derivations from which stabilized weights will be derived per subject per observation. Adjusted Kaplan–Meier curves were also presented along with hazard ratios and 95% confidence intervals, and adjusted p-values based on MSM and IPCW approaches. SAS proc PHREG procedure with counting process type of data input, which takes multiple observations per subject, was used as the final Cox model for OS for both MSM and IPCW approaches, where robust variance was used to accommodate covariance introduced by correlated longitudinal observations within each subjects and other extra variabilities due to departure from model assumptions.

PFS2 (defined as time from randomization to objective disease progression on next-line treatment or death from any cause, whichever occurs first) and time to next treatment (defined as the time from randomization to the first dose of the next line of antineoplastic therapy for any reason) were analyzed using similar statistical methods as PFS. For the exploratory ad-hoc analyses of OS according to next-line therapy, patients were analyzed according to treatment random assignment. Kaplan–Meier methodology was used, with comparisons between arms or groups carried out as described above. All statistics are descriptive for these analyses, as they were not prespeciﬁed, and the study was not powered to test for statistical signiﬁcance.

**SUPPLEMENTARY FIGURE**

## Supplementary Fig. S1. Mean change from study entry in EORTC QLQ-C30 global health status/QoL scores in TOURMALINE-MM3 (A) and TOURMALINE-MM4 (B).

**
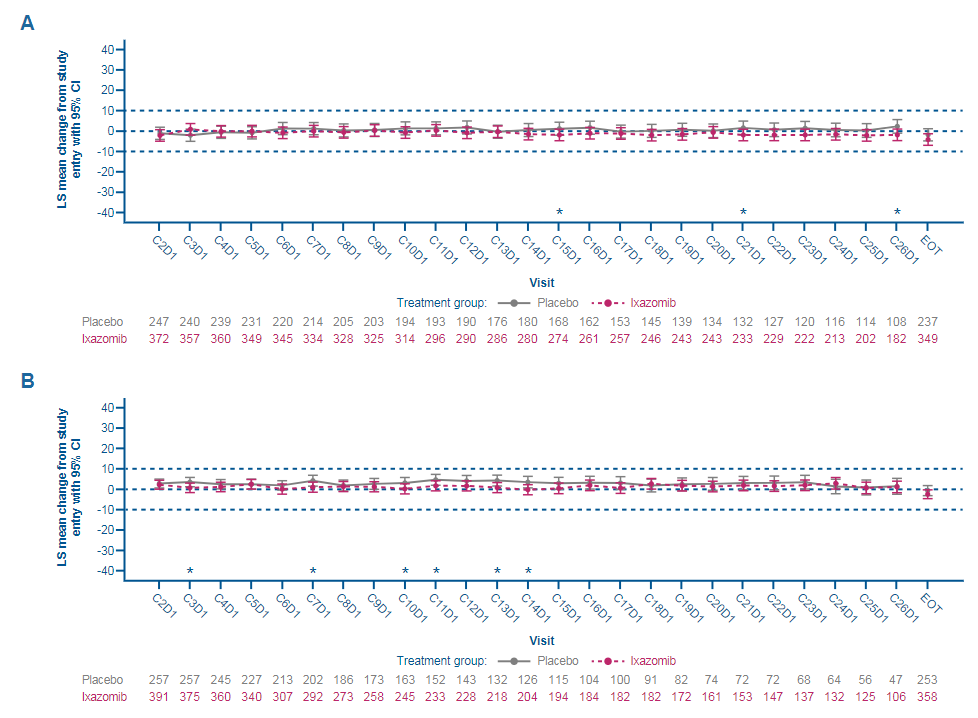
**

CI, confidence interval; EORTC QLQ-C30, European Organisation for Research and Treatment of Cancer Quality of Life Questionnaire – Core 30 Items; LS, least squares; QoL, quality of life.

**SUPPLEMENTARY TABLES**

## Supplementary Table S1. Demographics and baseline disease characteristics of patients in TOURMALINE-MM3.

| **Characteristic** | **Ixazomib**  **(N = 395)** | | | **Placebo**  **(N = 261)** |
| --- | --- | --- | --- | --- |
| **Median age (range), years** | 58 (52–63) | | | 60 (54–64) |
| **Male sex, n (%)** | 252 (64) | | | 162 (62) |
| **Race, n (%)** |  | | |  |
| White | 315 (80) | | | 213 (82) |
| Asian | 59 (15) | | | 36 (14) |
| Other/not reported | 21 (5) | | | 12 (5) |
| **Type of myeloma at initial diagnosis, n (%)** |  | | |  |
| Immunoglobulin G | 230 (58) | | | 149 (57) |
| Immunoglobulin A | 87 (22) | | | 60 (23) |
| Light chain | 66 (17) | | | 46 (18) |
| Other | 12 (3) | | | 6 (2) |
| **ISS disease stage at initial diagnosis, n (%)** | |  | |  |
| I | | 151 (38) | | 94 (36) |
| II | | 129 (33) | | 92 (35) |
| III | | 115 (29) | | 75 (29) |
| **ECOG PS at study entry, n (%)** | |  | |  |
| 0 | | 259 (66) | | 181 (69) |
| 1 | | 125 (32) | | 74 (28) |
| 2 | 11 (3) | | | 5 (2) |
| **Creatinine clearance at study entry, n (%)** | |  | |  |
| 30 to <60 ml/min | 38 (10) | | | 20 (8) |
| 60 to <90 ml/min | | 101 (26) | | 80 (31) |
| ≥90 ml/min | | 254 (64) | | 160 (61) |
| **High-risk cytogenetic abnormalities, n (%)** | | 61 (15) | | 54 (21) |
| **Induction regimen, n (%)** | |  | |  |
| Contained a PI | | 234 (59) | | 155 (59) |
| Contained an immunomodulatory drug | | 43 (11) | | 28 (11) |
| Contained a PI plus an immunomodulatory drug | | | 118 (30) | 78 (30) |
| **Response a after ASCT (study entry), n (%)** | | |  |  |
| CR | | | 80 (20) | 54 (21) |
| VGPR | | | 179 (45) | 115 (44) |
| PR | | | 84 (21) | 53 (20) |

ASCT, autologous stem cell transplant; CR, complete response; ECOG PS, Eastern Cooperative Oncology Group performance status; ISS, International Staging System; PI, proteasome inhibitor; PR, partial response; VGPR, very good partial response.

Reprinted from The Lancet, 393, Dimopoulos MA, Gay F, Schjesvold F, et al., Oral ixazomib maintenance following autologous stem cell transplantation (TOURMALINE-MM3): a double-blind, randomised, placebo-controlled phase 3 trial, 253-264, Copyright (2019), with permission from Elsevier.

## Supplementary Table S2. Patient demographics and baseline disease characteristics of patients in TOURMALINE-MM4.

| **Characteristic** | **Ixazomib**  **(N = 425)** | **Placebo**  **(N = 281)** |
| --- | --- | --- |
| **Age** |  |  |
| Median (range), years | 72 (42–89) | 73 (52–90) |
| <65 years, n (%) | 39 (9.2) | 29 (10.3) |
| ≥65 and <75 years, n (%) | 226 (53.2) | 142 (50.5) |
| ≥75 years, n (%) | 160 (37.6) | 110 (39.1) |
| **Male sex, n (%)** | 222 (52.2) | 155 (55.2) |
| **Race, n (%)** |  |  |
| White | 330 (77.6) | 227 (80.8) |
| Asian | 63 (14.8) | 39 (13.9) |
| Black or African American | 15 (3.5) | 5 (1.8) |
| **Type of myeloma at initial diagnosis, n (%)** |  |  |
| Immunoglobulin G | 252 (59.3) | 174 (61.9) |
| Immunoglobulin A | 102 (24.0) | 67 (23.8) |
| Light chain | 62 (14.6) | 36 (12.8) |
| Other^a^ | 9 (2.1) | 4 (1.4) |
| **ISS disease stage at initial diagnosis, n (%)** |  |  |
| I | 112 (26.4) | 66 (23.5) |
| II | 165 (38.8) | 114 (40.6) |
| III | 148 (34.8) | 101 (35.9) |
| **ECOG PS at study entry, n (%)** |  |  |
| 0 | 213 (50.1) | 147 (52.3) |
| 1 | 193 (45.4) | 120 (42.7) |
| 2 | 18 (4.2) | 14 (5.0) |
| **Frailty status, n (%)** |  |  |
| Fit | 172 (40.5) | 112 (39.9) |
| Unfit | 147 (34.6) | 98 (34.9) |
| Frail | 102 (24.0) | 68 (24.2) |
| **Creatinine clearance at study entry, n (%)** |  |  |
| 30 to <60 ml/min | 148 (34.8) | 104 (37.0) |
| 60 to <90 ml/min | 184 (43.3) | 108 (38.4) |
| ≥90 ml/min | 85 (20.0) | 65 (23.1) |
| **Cytogenetic features, n (%)** |  |  |
| High-risk cytogenetic abnormalities | 74 (17.4) | 48 (17.1) |
| Expanded high-risk cytogenetic abnormalities | 150 (35.3) | 91 (32.4) |
| **Elevated lactate dehydrogenase at study entry, n (%)** | 57 (13.4) | 38 (13.5) |
| **Evidence of lytic bone disease at study entry, n (%)** | 203 (47.8) | 141 (50.2) |
| **Induction regimen, n (%)** |  |  |
| Contained a PI | 351 (82.6) | 230 (81.9) |
| Contained bortezomib | 346 (81.4) | 228 (81.1) |
| Contained an immunomodulatory drug | 137 (32.2) | 94 (33.5) |
| Contained thalidomide | 92 (21.6) | 63 (22.4) |
| Contained lenalidomide | 47 (11.1) | 32 (11.4) |
| Contained a PI plus an immunomodulatory drug | 66 (15.5) | 44 (15.7) |
| **Common regimens (≥5% overall), n (%)** |  |  |
| VMP | 117 (27.5) | 88 (31.3) |
| VCd | 112 (26.4) | 75 (26.7) |
| VTd | 27 (6.4) | 14 (5.0) |
| Rd | 20 (4.7) | 16 (5.7) |
| CTd | 21 (4.9) | 14 (5.0) |
| **Response at study entry, n (%)** |  |  |
| CR | 96 (22.6) | 62 (22.1) |
| VGPR | 168 (39.5) | 112 (39.9) |
| PR | 161 (37.9) | 107 (38.1) |
| **Median time from start of induction to first maintenance dose (range), months** | 9.5 (5.6–15.0) | 9.4 (6.3–14.8) |

^a^Includes immunoglobulin D in five (1.2%) and two (0.7%) patients in the ixazomib and placebo arms, respectively; biclonal disease in two (0.5%) and one (0.4%) patients in the ixazomib and placebo arms, respectively; immunoglobulin M in two (0.5%) patients in the ixazomib arm and immunoglobulin E in one (0.4%) patient in the placebo arm.

CR, complete response; CTd, cyclophosphamide-thalidomide-dexamethasone; ECOG PS, Eastern Cooperative Oncology Group performance status; ISS, International Staging System; PI, proteasome inhibitor; PR, partial response; Rd, lenalidomide-dexamethasone; VCd, bortezomib-cyclophosphamide-dexamethasone; VGPR, very good partial response; VMP, bortezomib-melphalan-prednisone; VTd, bortezomib-thalidomide-dexamethasone.

Dimopoulos MA, Špička I, Quach H, et al: Ixazomib as postinduction maintenance for patients with newly diagnosed multiple myeloma not undergoing autologous stem cell transplantation: the phase III TOURMALINE-MM4 trial. J Clin Oncol 38(34):4030-4041, (2020), <https://doi.org/10.1200/JCO.20.02060>, with permission from Wolters Kluwer Health, Inc.

## Supplementary Table S3. TOURMALINE-MM4 safety summary.

| **n (%)** | **Ixazomib**  **(N = 426)** | **Placebo**  **(N = 276)** |
| --- | --- | --- |
| **Any TEAE** | 390 (92) | 227 (82) |
| Grade ≥3 | 166 (39) | 68 (25) |
| Treatment-related | 286 (67) | 113 (41) |
| Treatment-related grade ≥3 | 78 (18) | 12 (4) |
| **SAE** | 101 (24) | 48 (17) |
| Treatment-related SAE | 24 (6) | 3 (1) |
| **TEAEs resulting in dose modification of ixazomib/placebo^a^** | 210 (49) | 66 (24) |
| Resulting in dose reduction | 133 (31) | 14 (5) |
| Resulting in discontinuation | 62 (15) | 22 (8) |
| **On-study deaths^b^** | 11 (3) | 6 (2) |
| **Most common any-grade TEAEs^c^** |  |  |
| Nausea | 23 (8) | 119 (28) |
| Diarrhea | 35 (13) | 102 (24) |
| Vomiting | 13 (5) | 103 (24) |
| Upper respiratory tract infection | 32 (12) | 70 (16) |
| Peripheral sensory neuropathy | 25 (9) | 66 (15) |
| Back pain | 34 (12) | 62 (15) |
| Arthralgia | 31 (11) | 61 (14) |
| Fatigue | 28 (10) | 49 (12) |
| Pyrexia | 14 (5) | 46 (11) |

^a^Dose modification includes dose reduction, dose delay, and discontinuation of ixazomib or placebo. ^b^On-study deaths are defined as deaths that occurred within 30 days of the last dose of ixazomib or placebo.

^c^Occurring in ≥10% of patients in either treatment arm.

SAE, serious adverse event; TEAE, treatment-emergent adverse event.

## Supplementary Table S4. Overview of new primary malignancies (NPM) in TOURMALINE-MM3 and -MM4.

| **n (%)** | **Ixazomib** | **Placebo** |
| --- | --- | --- |
| **TOURMALINE-MM3** | N = 394 | N = 259 |
| **≥1 NPM** | 28 (7) | 21 (8) |
| **NPM type** |  |  |
| Hematologic | 6 (2) | 8 (3) |
| Non-hematologic | 12 (3) | 8 (3) |
| Non-hematologic (skin) | 12 (3) | 5 (2) |
| **TOURMALINE-MM4** | n=426 | n=276 |
| **≥1 NPM** | 34 (8) | 22 (8) |
| **NPM type** |  |  |
| Hematologic | 4 (<1) | 2 (<1) |
| Non-hematologic (melanoma) | 2 (<1) | 1 (<1) |
| Non-hematologic (non melanoma skin) | 10 (2) | 10 (4) |
| Non-hematologic (not skin) | 20 (5) | 13 (5) |

NPM, new primary malignancy.

**SUPPLEMENTARY REFERENCES**

1. Rajkumar SV, Harousseau JL, Durie B, Anderson KC, Dimopoulos M, Kyle R, et al. Consensus recommendations for the uniform reporting of clinical trials: report of the International Myeloma Workshop Consensus Panel 1. Blood 2011;117:4691-4695.
