# Supplementary material for: Final OS analyses from the TOURMALINE- MM3 and -MM4 RCTs of ixazomib maintenance in newly diagnosed multiple myeloma
Source: Blood Cancer J. 2025 Dec 4;16(1):15. doi: 10.1038/s41408-025-01411-9 (PMC12808303; doi:10.1038/s41408-025-01411-9)
Supplement: Supplementary file 2 — TOURMALINE-MM3 IRB and IEC Information [file 41408_2025_1411_MOESM2_ESM.docx]

#### IRB and IEC Information

The following table includes a list of IRBs and IECs used by investigators who received study drug, as well as any investigators who replaced them during the study.

| Principal Investigator | IRB or IEC | | | |
| --- | --- | --- | --- | --- |
| Bar, Daniel Oscar, MD (1902) | Comité Independiente de Etica en investigación clínica "Dr. Carlos A. Barclay Paraná 755, 6º A y B Ciudad Autónoma de Buenos Aires, Ciudad Autónoma de BuenosAires, C1107 Argentina  Comité de Docencia del Hospital J.B. Iturraspe Boulevard Carlos Pellegrini 3551 Santa Fe, Santa Fe S3000ADL Argentina  Comité Provincial de Bioética - Ministerio de Salud de la Provincia de Santa Fé Boulevard Gálvez 1563 Santa Fé, Santa Fe, 3000 Argentina  Administración Nacional de Medicamentos Alimentos y Tecnología Médica (ANMAT) Avenida De Mayo 869 Montserrat, Ciudad Autónoma de BuenosAires, C1084AAD Argentina | | | |
| Basso, Alfredo Carlos, MD (1904) | Comité Independiente de Etica en investigación clínica "Dr. Carlos A. Barclay Paraná 755, 6º A y B Ciudad Autónoma de Buenos Aires,  Ciudad Autónoma de BuenosAires, C1107 Argentina  Comité de Docencia e Investigación del Sanatorio Parque de Rosario Boulevard Nicasio Oroño 860 Rosario, Santa Fe, S2000DSU Argentina  Comité Provincial de Bioética - Ministerio de Salud de la Provincia de Santa Fé Boulevard Gálvez 1563 Santa Fé, Santa Fe, 3000 Argentina  Administración Nacional de Medicamentos Alimentos y Tecnología Médica (ANMAT) Avenida De Mayo 869 Montserrat, Ciudad Autónoma de BuenosAires, C1084AAD Argentina | | | |
| Fantl, Dorotea Beatriz Eugenia, MD (1905) | Comité de Ética de Protocolos de Investigación Juan Domingo Perón 4190/4192Buenos Aires, Ciudad Autónoma de BuenosAires, 1181 Argentina  Administración Nacional de Medicamentos Alimentos y Tecnología Médica (ANMAT) Avenida De Mayo 869 Montserrat, Ciudad Autónoma de BuenosAires, C1084AAD Argentina | | | |
| He, Simon, MD (2902) | Therapeutic Goods Administration 136 Narrabundah Lane Symonston, ACT, 2609 Australia  Hunter New England Research Ethics and Governance Unit LOCKED BAG 1 New Lambton, New South Wales, 2305 Australia   Austin Health Human Research Ethics Committee 145 Studley Road Heidelberg, Vic, 3084 Australia | | | |
| Horvath, Noemi, MD (2903) | Hunter New England Research Ethics and Governance Unit LOCKED BAG 1 New Lambton, New South Wales, 2305 Australia | | | |
| Lee, Cindy, MD (2904) Gray, James, MD | Therapeutic Goods Administration 136 Narrabundah Lane Symonston, ACT, 2609 Australia  Hunter New England Research Ethics and Governance Unit LOCKED BAG 1 New Lambton, New South Wales, 2305 Australia | | | |
| Rowlings, Philip, MD (2905) | Therapeutic Goods Administration 136 Narrabundah Lane Symonston, ACT, 2609 Australia  Hunter New England Research Ethics and Governance Unit LOCKED BAG 1 New Lambton, New South Wales, 2305 Australia | | | |
| Taylor, Kerry, MBBS (2906) | Bellberry Human Research Ethics Committee 123 Glen Osmond Road Eastwood, South Australia, 5063 Australia  Therapeutic Goods Administration 136 Narrabundah Lane Symonston, ACT, 2609 Australia | | | |
| Spencer, Andrew, MBBS (2907) | Hunter New England Research Ethics and Governance Unit LOCKED b 1 New Lambton, New South Wales, 2305 Australia  The Alfred Hospital Ethics Committee Commercial Road Melbourne, Vic, 3004 Australia  Melbourne Health Human Research Ethics Committee Grattan Street, Human Research Ethics Committee Directorate, Post Office, 6 East, Main Building Parkville, Victoria, 3050 Australia  Therapeutic Goods Administration 136 Narrabundah Lane Symonston, ACT, 2609 Australia | | | |
| Cochrane, Tara, MD (2908) | Hunter New England Research Ethics and Governance Unit LOCKED BAG 1 New Lambton, New South Wales, 2305 Australia  Therapeutic Goods Administration 136 Narrabundah Lane Symonston, ACT, 2609 Australia | | | |
| Kwok, Fiona, MBBS (2909) | Therapeutic Goods Administration 136 Narrabundah Lane Symonston, ACT, 2609 Australia  Hunter New England Research Ethics and Governance Unit LOCKED BAG 1 New Lambton, New South Wales, 2305 Australia | | | |
| Ramanathan, Sundreswran (Sundra), MBBS (2910) | Therapeutic Goods Administration 136 Narrabundah Lane Symonston, ACT, 2609 Australia  Hunter New England Research Ethics and Governance Unit LOCKED BAG 1 New Lambton, New South Wales, 2305 Australia | | | |
| Agis, Hermine, PD (3901) Zojer, Niklas, PD | Ethikkommission für das Bundesland Salzburg Michael-Pacher-Straße  Salzburg 5020 Austria  Ethikkommission der Medizinischen Universität Wien Borschkegasse 8b/ E 06, Dienstzimmergebäude, BT 68 Wien, 1090 Austria | | | |
| Zojer, Niklas, MD (3903) | Ethikkommission für das Bundesland Salzburg Michael-Pacher-Straße  Salzburg 5020 Austria  Ethikkommission der Stadt Wien Thomas-Klestil-Platz 8 Wien, 1030 Austria | | | |
| Kentos, Alain, MD (4902) | Etische Comité UZ Gent Corneel Heymanslaan 10 Gent, Oost-Vlaanderen, 9000 Belgium  Federal Agency for Medicines and Health Products Avenue Galilée 5/03 Brussels, 1210 Belgium | | | |
| Offner, Fritz, MD (4903) | Etische Comité UZ Gent  Corneel Heymanslaan 10  Gent, Oost-Vlaanderen, 9000  Belgium   Federal Agency for Medicines and Health Products  Avenue Galilée 5/03  Brussels, 1210  Belgium | | | |
| Van Droogenbroeck, Jan, MD (4904) | | | Etische Comité UZ Gent  Corneel Heymanslaan 10  Gent, Oost-Vlaanderen, 9000  Belgium  Federal Agency for Medicines and Health Products  Avenue Galilée 5/03  Brussels, 1210  Belgium   AZ Sint-Jan AV - Ethisch Comité Ruddershove 10 Brugge, West-Vlaanderen, 8000 Belgium | |
| Wu, Ka Lung, MD (4905) | | | Etische Comité UZ Gent  Corneel Heymanslaan 10  Gent, Oost-Vlaanderen, 9000  Belgium   Federal Agency for Medicines and Health Products  Avenue Galilée 5/03  Brussels, 1210  Belgium   Commissie voor Medische Ethiek - ZNA Middelheim Lindendreef 1, AZ Middelheim Antwerpen, 2020 Belgium | |
| Aparecida Martinez, Gracia, MD (5905) | | | Comissão Nacional de Ética em Pesquisa- CONEP  SRTV 701, Via W 5 Norte, Lote D – Edíficio PO 700, 3º andar, Asa Norte  Brasília, Distrito Federal, 70719-040  Brazil  Comitê de Ética em Pesquisa da Faculdade de Medicina da Universidade de São Paulo/ CAPPesq Avenida Doutor Arnaldo, 455, 1º andar São Paulo, 01246-903 Brazil  Agência Nacional de Vigilância Sanitária Setor de Indústria e Abastecimento Trecho 5, Trecho 5, Térreo, Área Especial 57 Brasília, Distrito Federal, 71205-050 Brazil | |
| Araújo, Sérgio Schustcrschitz da Silva, MD (5915) Fantl, Dorotea, MD | | | Comissão Nacional de Ética em Pesquisa- CONEP SRTV 701, Via W 5 Norte, Lote D – Edíficio PO 700, 3º andar, Asa Norte Brasília, Distrito Federal, 70719-040 Brazil  Comitê de Ética em Pesquisa em Seres Humanos da Universidade Federal de Minas Gerais - COEP UFMG Avenida Antonio Carlos, 6627 Pampulha Belo Horizonte, MG 31270-901 Brazil  Agência Nacional de Vigilância Sanitária Setor de Indústria e Abastecimento Trecho 5, Trecho 5, Térreo, Área Especial 57 Brasília, Distrito Federal, 71205-050 Brazil | |
| Capra, Marcelo Eduardo Zanella, MD (5910) | | | Comissão Nacional de Ética em Pesquisa- CONEP SRTV 701, Via W 5 Norte, Lote D – Edíficio PO 700, 3º andar, Asa Norte Brasília, Distrito Federal, 70719-040 Brazil  Comitê de Ética em Pesquisa - Hospital Mãe de DeusRua José de Alencar, 286Porto Alegre, RS 90880-480 Brazil  Agência Nacional de Vigilância Sanitária Setor de Indústria e Abastecimento Trecho 5, Trecho 5, Térreo, Área Especial 57 Brasília, Distrito Federal, 71205-050 Brazil | |
| Maiolino, Angelo, MD (5901) | Comissão Nacional de Ética em Pesquisa- CONEP, SRTV 701 Via W 5 Norte, Lote D – Edíficio PO 700, 3º andar, Asa NorteBrasília, Distrito Federal, 70719-040 Brazil  Comitê de Ética em Pesquisa do Hospital Universitário Clementino Fraga Filho  Rua Professor Rodolpho Paulo Rocco, 255 Cidade Universitária – Ilha do Fundão Rio de Janeiro, RJ 21941-913 Brazil  Comitê de Ética em Pesquisa Clínica do Hospital Universitário Clementino Fraga Filho Hospital Universitário Pedro Ernesto Av. Vinte e Oito de Setembro, 77- 2º andar Vila Isabel Rio de Janeiro, 20551-030 Brazil  Agência Nacional de Vigilância Sanitária Setor de Indústria e Abastecimento Trecho 5, Trecho 5, Térreo, Área Especial 57 Brasília, Distrito Federal, 71205-050 Brazil | | |  |
| Peters, Lygia Goretti, MD (5909) Bruggemann, MD (Former PI) Zanella, Karla Richter, MD | Comissão Nacional de Ética em Pesquisa- CONEP SRTV 701, Via W 5 Norte, Lote D – Edíficio PO 700, 3º andar, Asa Norte Brasília, DF 70719-040 Brazil  Comitê de Ética em Pesquisa do Centro de Pesquisas Oncológicas – CEPON Rodovia Ademar Gonzaga Km 0,5 Florianópolis, Santa Catarina 88034-000 Brazil  Agência Nacional de Vigilância Sanitária Setor de Indústria e Abastecimento Trecho 5, Trecho 5, Térreo, Área Especial 57 Brasília, Distrito Federal, 71205-050 Brazil | | |  |
| Pavlicek, Petr, MD (12901) Evžen, Gregora, MD | Etická komise Fakultní nemocnice Královské Vinohrady Šrobárova 50 Praha 10 100 34 Czech Republic  does not appear in Submissions tab | | |  |
| Hájek, Roman, MD (12902) | Etická komise Fakultní nemocnice Ostrava17. listopadu 1790 Ostrava, Moravskoslezský kraj 708 52 Czech Republic | | |  |
| Maisner, Vladimír, MD (12903) | Etická komise Fakultní nemocnice Hradec Králové Sokolska 581 Hradec Králové 500 05 Czech Republic  MEC: Etická komise Fakultní nemocnice Ostrava  17. listopadu 1790 Ostrava - Poruba 708 52 Czech Republic  Eticka komise Fakultni nemocnice Kralovske Vinohrady Srobarova 50 Praha 10, 100 34 Czech Republic | | |  |
| Pour, Ludĕk, MUDr, PhD, MUDr (12904) | Etická komise Fakultní nemocnice Brno Jihlavská 340/20Brno 625 00 Czech Republic  MEC: Etická komise Fakultní nemocnice Ostrava 17. listopadu 1790 Ostrava - Poruba 708 52 Czech Republic | | |  |
| Pika, Tomas, MD (12905) Ščudla, Vlastimil, CSc, MUDr | Etická komise Fakultní nemocnice Olomouc I. P. Pavlova 185/6 Olomouc 779 00 Czech Republic  MEC: Etická komise Fakultní nemocnice Ostrava 17. listopadu 1790 Ostrava - Poruba 708 52 Czech Republic | | |  |
| Špička, Ivan, CSc, MUDr (12906) | Etická komise Všeobecné Fakultní nemocnice v Praze Na Bojišti 1771/1 Praha 2, Praha, hlavní mesto, 120 00 Czech Republic  MEC: Etická komise Fakultní nemocnice Ostrava 17. listopadu 1790 Ostrava - Poruba 708 52 Czech Republic | | |  |
| Abildgaard, Niels, DMSc (13901) Andersen, Niels, MD (13902) Jensen, Bo Amdi, MD (13903) Helleberg, Carsten, MD (13904) Plesner, Torben, MD (13905) Helleberg, Carsten, MD (13906) Salomo, Morten, MD Vangsted, Annette Juul Svirskaite, Asta (13907)  Gregersen, Henrik, MD | | Lægemiddelstyrelsen Axel Heides Gade 1 Copenhagen, Hovedstaden, DK-2300 Denmark  De Videnskabsetiske Komiteér for Region Syddanmark Damhaven 12, Regionshuset, 3. Sal Vejle, South Denmark 7100 Denmark | | |
| Nogai, Axel, MD (19901) Blau, Igor, MD Janjetovic, Snjezana, MD (19910) Schmidt-Hieber, Martin, MD Fuhrmann, Stephan, MD | | Ethikkommission der Medizinischen Fakultät Heidelberg Alte Glockengießerei 11/1 Heidelberg 69115 Germany  Landesamt für Gesundheit und Soziales Berlin (LAGeSo)_Landesbehörde Turmstr. 21  Berlin, 10559 Germany | | |
| Goldschmidt, Hartmut, MD (19903) | | Regierungspräsidium Tübingen Dienstsitz Tübingen Konrad-Adenauer-Str. 20 Tübingen, Baden-Württemberg, 72072 Germany  Regierungspräsidium Karlsruhe Markgrafenstr. 46 Karlsruhe, Baden-Württemberg, 76133 Germany  Ethikkommission der Medizinischen Fakultät Heidelberg Alte Glockengießerei 11/1 Heidelberg 69115 Germany | | |
| Weisel, Katja, MD (19905) Hansen, Timon, MD Binder, Mascha, MD Schieferdecker, Aneta, MD  Salwender, Hans-Jurgen, MD (19909) | | Ethikkommission der Medizinischen Fakultät Heidelberg Alte Glockengießerei 11/1 Heidelberg 69115 Germany  Ethik-Kommission der Ärztekammer Hamburg Weidestr. 122 b Hamburg, 22083 Germany  Behörde für Gesundheit und Verbraucherschutz der Freien und Hansestadt Hamburg Billstraße 80, Abteilung V4, Pharmaziewesen und Medizinprodukte, Behörde für Gesundheit und Verbraucherschutz der Freien und Hansestadt Hamburg Hamburg, 20539 Germany | | |
| Kull, Miriam, MD (19906) Langer, Christian, MD Teleanu, Veronica | | Regierungspräsidium Tübingen Dienstsitz Tübingen Konrad-Adenauer-Str. 20 Tübingen, Baden-Württemberg, 72072 Germany  Regierungspräsidium Karlsruhe Markgrafenstr. 46 Karlsruhe, Baden-Württemberg, 76133 Germany  Ethikkommission der Universität Ulm Helmholtzstraße 20 Ulm, 89081 Germany | | |
| Munder, Markus, MD (19907) Hoffman, Martin, MD (19922) | | Landesamt für Soziales, Jugend und Versorgung Dienstsitz Trier Moltkestraße 19, Referat 54.4 Trier, 54292 Germany  Ethikkommission der Medizinischen Fakultät Heidelberg Alte Glockengießerei 11/1 Heidelberg 69115 Germany  Ethik-Kommission der Landesärztekammer Rheinland-Pfalz Deutschhausplatz 3 Mainz, Rheinland-Pfalz, 55116 Germany | | |
| Rollig, Christoph, MD (19908) | | Landesdirektion Sachsen Braustrasse 2, Dienststelle Leipzig Referat 24.2 L, Pharmazie, GMP-Inspektorat Leipzig, 4107 Germany  Ethikkommission der Medizinischen Fakultät Heidelberg Alte Glockengießerei 11/1 Heidelberg 69115 Germany  Ethik-Kommission der Medizinischen Fakultät "Carl Gustav Carus" der Technischen Universität Dresden Fetscherstraße 74 Dresden, 01307 Germany | | |
| Besemer, Britta, MD (19911) Weisel, Katja, MD | | Ethik-Kommission der Medizinischen Fakultät der Eberhard-Karls-Universität Gartenstraße 47 Tübingen, Baden-Württemberg, 72074 Germany  Ethikkommission der Medizinischen Fakultät Heidelberg Alte Glockengießerei 11/1 Heidelberg 69115 Germany  Regierungspräsidium Karlsruhe Markgrafenstr. 46 Karlsruhe, Baden-Württemberg, 76133 Germany  Regierungspräsidium Tübingen Dienstsitz Tübingen Konrad-Adenauer-Str. 20 Tübingen, Baden-Württemberg, 72072 Germany | | |
| Durig, Jan, MD (19913) | | Gesundheitsamt der Stadt Düsseldorf Willi-Becker-Allee 10, Zentrales Inspektorat für klinische Prüfstellen in Nordrhein-Westfalen Düsseldorf, 40227 Germany  Ethikkommission der Medizinischen Fakultät Heidelberg Alte Glockengießerei 11/1 Heidelberg 69115 Germany  Ethik-Kommission der Medizinischen Fakultät der Universität Duisburg-Essen Robert-Koch-Straße 9-11 Essen, Nordrhein-Westfalen, 45147 Germany | | |
| Zeis, Matthias, MD (19914) | | Behörde für Gesundheit und Verbraucherschutz der Freien und Hansestadt Hamburg Billstraße 80, Abteilung V4, Pharmaziewesen und Medizinprodukte, Behörde für Gesundheit und Verbraucherschutz der Freien und Hansestadt Hamburg Hamburg, 20539 Germany  Ethikkommission der Medizinischen Fakultät Heidelberg Alte Glockengießerei 11/1 Heidelberg 69115 Germany | | |
| Klein, Sefan, MD (19915) | | Ethik-Kommission II der Universität Heidelberg Universitätsklinikum Mannheim Theodor-Kutzer-Ufer 1-3 Mannheim, 68167 Germany  Ethikkommission der Medizinischen Fakultät Heidelberg Alte Glockengießerei 11/1 Heidelberg 69115 Germany  Regierungspräsidium Karlsruhe Markgrafenstr. 46 Karlsruhe, Baden-Württemberg, 76133 Germany  Regierungspräsidium Tübingen Dienstsitz Tübingen Konrad-Adenauer-Str. 20 Tübingen, Baden-Württemberg, 72072 Germany | | |
| Reimer, Peter, MD (19916) | | Gesundheitsamt der Stadt Düsseldorf Willi-Becker-Allee 10, Zentrales Inspektorat für klinische Prüfstellen in Nordrhein-Westfalen Düsseldorf, 40227 Germany  Ethikkommission der Medizinischen Fakultät Heidelberg Alte Glockengießerei 11/1 Heidelberg 69115 Germany | | |
| Schmidt, Christian, MD (19917) Ostermann, Helmut, MD | | Regierung von Oberbayern Maximilianstraße 39, Sachgebiet 53.2 Pharmazie, Oberbayern, Niederbayern, Schwaben München, 80538 Germany  Ethikkommission der Medizinischen Fakultät Heidelberg Alte Glockengießerei 11/1 Heidelberg 69115 Germany  Ethikkommission der Med. Fakultät der LMU München Pettenkoferstr. 8a München, 80336 Germany | | |
| Scheid, Christof, MD (19918) | | Ethik-Kommission der Medizinischen Fakultät der Universität zu Köln Kerpenerstr. 62, Gebäude 5 Köln, 50937 Germany  Ethikkommission der Medizinischen Fakultät Heidelberg Alte Glockengießerei 11/1 Heidelberg 69115 Germany  Gesundheitsamt der Stadt Düsseldorf Willi-Becker-Allee 10, Zentrales Inspektorat für klinische Prüfstellen in Nordrhein-Westfalen Düsseldorf, 40227 Germany | | |
| Mayer, Karin, MD (19921) Janzen, Viktor, MD | | Ethik-Kommission Bonn der Medizinischen Fakultät der Rheinisch Friedrich-Wilhelms-Universität Sigmund-Freud-Str. 25 Bonn, Nordrhein-Westfalen, 53127 Germany  Ethik-Kommission der Ärztekammer Westfalen-Lippe und der Medizinischen Fakultät der WWU Munster Gartenstrasse 210 – 214 Münster, Nordrhein-Westfalen, 48147 Germany  Ethikkommission der Medizinischen Fakultät Heidelberg Alte Glockengießerei 11/1 Heidelberg 69115 Germany  Gesundheitsamt der Stadt Düsseldorf Willi-Becker-Allee 10, Zentrales Inspektorat für klinische Prüfstellen in Nordrhein-Westfalen Düsseldorf, 40227 Germany | | |
| Sosada, Markus (19923) Kirchner, Hartmut, MD | | Staatliches Gewerbeaufsichtsamt Hannover Am Listholze 74, Dez. 24 - Arzneimittelrecht, Hannover, 20177 Germany  Ethikkommission der Medizinischen Fakultät Heidelberg Alte Glockengießerei 11/1 Heidelberg 69115 Germany | | |
| Dimopoulos, Meletios –  Athanasios, MD (20901) | | National Ethics Committee (EED) 284, Mesogion Avenue Cholargos Athens, Atikki 15562 Greece  Alexandra Hospital 80 Vasilissis Sofias Athens, 11528 Greece | | |
| Delimpasi, Sosana, MD (20902) | | National Ethics Committee (EED) 284, Mesogion Avenue Cholargos Athens, Atikki 15562 Greece  Evangelismos Hospital of Athens 45-47 Ipsilantou, Intensive Care Unit 1, 3rd floor Athens, 10676 Greece | | |
| Kyrtsonis, Marie Christine, MD (20903) | | National Ethics Committee (EED) 284, Mesogion Avenue Cholargos Athens, Atikki 15562 Greece  Laiko General Hospital of Athens 17 Agiou Thoma str Athens, Atiki, 11527 Greece | | |
| Sakellari, Ioanna, MD (20904) Anagnostopoulos, Achilleas, MD | | National Ethics Committee (EED) 284, Mesogion Avenue Cholargos Athens, Atikki 15562 Greece  Georgios Papanikolaou General Hospital of Thessaloniki Exohi Thessaloniki, 57010 Greece | | |
| Borbényi, Zita, MD (22904) Egyed, Miklós, MD (22903) Illés, Árpád, MD (22902) Mikala, Gábor, MD (22905) Masszi, Tamás, MD  Nagy, Gyorgy, MD (22901) Demeter, Judit, MD | | Egészségügyi Tudományos Tanács Klinikai Farmakológiai Etikai Bizottság  Arany János utca 6-8 Budapest 1051 Hungary  Orszagos Gyogyszereszeti es Elelmezes-egeszsegugyi Intezet 33 Szabolcsutca Budapest 1035 Hungary | | |
| Dally, Najib, MD (27903) | | Ziv Medical Center Local EC Ziv Medical Center, Endocrinology Unit, POB 1008 Safed 13100 Israel | | |
| Horowitz, Netanel Avraham, MD (27904) | | Rambam Medical Center Institutional Helsinki Committee 8 Haaliya Hashniya Street, Infectious Diseases Unit, P.O. Box 9602Haifa 31096 Israel  Rambam Medical Center Ethics Committee 8 Haaliya Hashniya Street, Bat Galim Haifa 31096 Israel | | |
| Koren-Michowitz, Maya, MD (27905) Odit, Gutwein | | Shamir Medical Center Assaf Harofeh ECBeer Yaakov 70300 Zerifin 70300 Israel | | |
| Nemets, Anatoly, MD (27906) Lugassy, Gilles, MD | | Barzilai Medical Center Local EC 2 Hahistadrout Street Ashkelon 78278 Israel | | |
| Cohen, Amos M, MD (27907)  Vaxman, Iuliana, MD Pasvolski, Oren, MD | | Institutional Helsinki Committee Rabin Medical Center - Beilinson Campus 39 Jabotinsky Street Petach Tikva 49100 Israel | | |
| Shvetz, Olga, MD (27908) | | Institutional Helsinki Committee Kaplan Medical Center, PO Box 1 Rehovot 76100 Israel | | |
| Trestman, Svetlana, MD (27909) | | Institutional Helsinki Committee Tel Aviv Sourasky Medical Center Tel Aviv 64239 Israel | | |
| Ronson,Aaron, MD (27910) Ruchlemer, Rosa, MD | | Institutional Helsinki Committee Shaare Zedek Medical Center Ta Do'ar 3235, 12 Shmuel' Beit Jerusalem, Yerushalayim, 90000 Israel | | |
| Nagler, Arnon, MD (27911) | | Institutional Helsinki Committee Chaim Sheba Medical Center Tel-Hashomer Ramat-Gan 52621 Israel | | |
| Tadmor, Tamar, MD (27912) Attias, Dina, MD | | Institutional Helsinki Committee Bnai Zion Medical Center 47 Eliyahu Golomb Street., Post Office Box 4940 Haifa 31048 Israel | | |
| Rouvio, Ory, MD (27913) | | Institutional Helsinki Committee Soroka University Medical Center, Post Office Box 151, Shderot Yitshak Rager Be'er Sheva 84101 Israel | | |
| Preis, Meir, MD (27914) | | Institutional Helsinki Committee Lady Davis Carmel Medical Center 7 Michal Street Haifa 34362 Israel | | |
| Preis, Meir, MD (27914) | | Institutional Helsinki Committee Lady Davis Carmel Medical Center 7 Michal Street Haifa 34362 Israel | | |
| Mina, Roberto, MD (28901) Gay, Francesca, MD Palumbo, Antonio, MD  Cavo, Michele, MD (28903)  Garzia, Mariagrazia (28905)  De Rosa, Luca, MD  Pietrantuono, Giuseppe (28909) Musto, Pellegrino, MD  Cafro, Anna Maria, MD (28910)  Tosi, Patrizia, MD (28911)  Offidani, Massimo, MD (28912)  Mangiacavalli, Silvia, MD (28913) Corso, Alessandro, MD  Rossi, Giuseppe, MD (28914)  Liberati, Anna Marina, MD (28916)  Antonioli, Elisabetta, MD (28917) Bosi, Alberto, MD | | Comitato Etico Milano Area C A.O. Ospedale Niguarda Ca' Granda Piazza Ospedale Maggiore 3 Milan, Milan 20162 Italy | | |
|  | |  | | |
|  | |  | | |
|  | |  | | |
|  | |  | | |
|  | |  | | |
|  | |  | | |
|  | |  | | |
|  | |  | | |
|  | |  | | |
| Chang Ki Min, MD (29905) | | The Catholic University of Korea, Seoul St. Mary's Hospital IRB 222 Banpo daero Seocho gu, 9F Annex Bldg Seoul, 06591  Republic of Korea | | |
| Yoon, Dok Hyun, PhD (29907) Suh, Cheolwon, MD | | Asan Medical Center IRB Seoulasanbyeongweon, 88 Olympic-ro 43-gil, Pungnap-Dong  Songpa-Gu, Seoul Teugbyeolsi, 05505 Republic of Korea | | |
| Deog-Yeon Jo, MD(29902) | | Chungnam National University Hospital IRB 282, Munhwa-ro, Jung-Gu Daejeon, 35015 Republic of Korea | | |
| Eom, Hyeon Seok, MD (29901) | | National Cancer Center Institutional Review Board 323 llsan-ro, llsandong-gu  Goyang-si, Gyeonggido, 10408 Republic of Korea | | |
| Lee, Jae Hoon, PhD (29904) | | Gachon University Gil Medical Center Institutional Review Board Gacheondaehakgyo Gilbyeongweon, 1198 Guwol-Dong Namdong-Gu, Incheon Gwang'yeogsi, 21565 Republic of Korea | | |
| Kim, Jin Seok MD(29903) | | Severance Hospital, Yonsei University Health System IRB Yeonsedaehakgyo, 50-1 Yonsei-Ro, Sinchon-Dong  Seodaemun-Gu, Seoul Teugbyeolsi, 03722  Republic of Korea | | |
| Kim, Kihyun, MD (29908) | | Samsung Medical Center IRB 81, Irwon-ro, Gangnam-gu Seoul, 06351 Republic of Korea | | |
| Yoon, SungSoo MD (29906) | | Seoul National University Hospital IRB 101 Daehak-ro, Yeongeon-Dong Jongno-Gu, Seoul Teugbyeolsi, 03080 Republic of Korea | | |
| Zweegman, Sonja, MD (36901) | | METC VUmc Van Der Boechorststraat Amsterdam, Noord-Holland 1081 BT The Netherlands  CCMO Parnassusplein 5, Bevoegde Instantie Den Haag, Zuid-Holland, 2511 VX Netherlands  Raad van Bestuur VU Medisch Centrum De Boelelaan 1117 Amsterdam, Noord-Holland, 1081 HV Netherlands | | |
| Levin, Mark-David, MD (36903) | | Raad van Bestuur Albert Schweitzer Ziekenhuis Albert Schweitzerplaats 25 Dordrecht, Zuid-Holland, 3318 AT Netherlands  METC VUmc Van Der Boechorststraat Amsterdam, Noord-Holland 1081 BT The Netherlands  CCMO Parnassusplein 5, Bevoegde Instantie Den Haag, Zuid-Holland, 2511 VX Netherlands | | |
| Roeloffzen, Wilfred, MD (36904) De Waal, Elisabeth, MD Vellenga, Edo | | Raad van Bestuur Universitair Medisch Centrum Groningen Hanzeplein 1 Groningen, Groningen, 9713 GZ Netherlands  METC VUmc Van Der Boechorststraat Amsterdam, Noord-Holland 1081 BT The Netherlands  CCMO Parnassusplein 5, Bevoegde Instantie Den Haag, Zuid-Holland, 2511 VX Netherlands | | |
| Minnema, Monique, MD (36905) | | Raad van Bestuur Universitair Medisch Centrum Utrecht Heidelberglaan 100 Utrecht, Utrecht, 3584 CX Netherlands  METC VUmc Van Der Boechorststraat Amsterdam, Noord-Holland 1081 BT The Netherlands  CCMO Parnassusplein 5, Bevoegde Instantie Den Haag, Zuid-Holland, 2511 VX Netherlands | | |
| Haukäs, Einar, MD (38905) Waage, Anders, MD (38903) Schjesvold, Fredrik, MD (38901) | | Regional komité for medisinsk og helsefaglig forskningsetikk, Sør-Øst-Norge (REK Sør-Øst)  Gullhaugveien 1-3 Oslo, N-0484 Norway | | |
| Grosicki, Sebastian, PhD (42901) Krzanowski, Jacek, MD (42902) Pluta, Andrzej, MD  Robak, Tadeusz, MD (42903) | | Komisja Bioetyczna Slaskiej Izby Lekarskiej w Katowicach Grazynskiego 49 Katowice, Slaskie, 40-126 Poland  Urzad Rejestracji Produktów Leczniczych, Wyrobów Medycznych i Produktów Biobójczych AL.Jerozolimskie 181C Warszawa, Mazowieckie, 02-222 Poland | | |
| Marques, Herlander, MD (43901) Bergantim, Rui Filipe Cordeiro, MD (43903) Guimaraes, Jose Eduardo, MD Leite, Luis, MD (43905) | | CEIC - Comissão de Ética para a Investigação Clínica Avenida do Brasil, 53, Pavilhão 17-A Lisboa, Lisboa, 1749-004 Portugal | | |
| Chng, Wee Joo, MD (48901) | | Domain Specific Review Board Nexus @ One-North (South Tower), No.3 Fusionopolis Link, #03-08 Singapore, Singapore 138543 Singapore | | |
| Goh, Yeow Tee, MBBS (48902) | | Singhealth Centralised Institutional Review Board 7 Hospital Drive, Singhealth Office Of Research, Blk A, #03-01, Singhealth Research Facilities Singapore, Singapore, 169611 Singapore | | |
| McDonald, Andrew, MBBS  (50901) | | South African Medical Association Research Ethics Committee (SAMAREC) Nossob Street, Block F, Castle Walk Corporate Park, The South African Medical Association  Pretoria, Gauteng 0153 South Africa  South African Health Products Regulatory Authority (SAHPRA) Cnr Andries And Bloed Streets, Civitas Building, Department Of Health, Room NG 90, South African Medicines Control Council Pretoria, Gauteng 0001 South Africa | | |
| Rapoport, Bernado Leon, MD (50903),  Alvarez Rivas, Miguel Angel, PhD (51901) Carrillo Cruz, Estrella, MD (51906) Serrano, Chacon,Maria Dolores, MD Martin Sanchez, Jesus, MD | | CEIC Hospital Puerta de Hierro Majadahonda C/ Joaquín Rodrigo, 2, Secretaría Técnica del CEIC, Pasillo Unidades Administrativas (planta 1 / Peines 6-7), Entrada Por Laboratorios-banco De Sangre Majadahonda, Madrid 28222 Spain  CEIC de Andalucia (CCEIBA) Avenida de la Innovación s/n Sevilla, Andalucia, 41020 Spain | | |
| Lopez de Ia Guia, Ana, MD (51914) | | CEIC Hospital Universitario La Paz Paseo de la Castellana, 261, Hospital General - Comité Ético de Investigación Clínica, Planta 8 Madrid, Madrid, 28046 Spain  CEIC Hospital Puerta de Hierro Majadahonda  C/ Joaquín Rodrigo, 2, Secretaría Técnica del CEIC, Pasillo Unidades Administrativas (planta 1 / Peines 6-7), Entrada Por Laboratorios-banco De Sangre Majadahonda, Madrid 28222 Spain | | |
| Amor, Adrian, MD (51913) | | CEIC Hospital Universitario de la Princesa Calle Diego de León, 62, Fundación para la Investigación Biomédica, 1ª Planta, Secretaría Técnica del CEIC Madrid, Madrid, 28006 Spain  CEIC Hospital Puerta de Hierro Majadahonda  C/ Joaquín Rodrigo, 2, Secretaría Técnica del CEIC, Pasillo Unidades Administrativas (planta 1 / Peines 6-7), Entrada Por Laboratorios-banco De Sangre Majadahonda, Madrid 28222 Spain | | |
| Encinas, Cristina, MD (51912) Gayoso Cruz, Jorge, MD | | CEIC Hospital Puerta de Hierro Majadahonda  C/ Joaquín Rodrigo, 2, Secretaría Técnica del CEIC, Pasillo Unidades Administrativas (planta 1 / Peines 6-7), Entrada Por Laboratorios-banco De Sangre Majadahonda, Madrid 28222 Spain  CEIC Hospital General Universitario Gregorio Marañon Calle Doctor Esquerdo, 46, Oficina Tecnica CEIC-A1, Pabellon de Gobierno - Planta baja, Fundacion para la Investigacion Biomedica Madrid, Madrid, 28007 Spain | | |
| Perez de Oteyza, Jaime, MD (51911) | | CEIC Grupo Hospital de Madrid Avenida Monteprincipe, 25, Secretaria del Comite Etico de Investigacion Clinica, Grupo Hospitales de Madrid Boadilla del Monte, Madrid, 28660 Spain  CEIC Hospital Puerta de Hierro Majadahonda  C/ Joaquín Rodrigo, 2, Secretaría Técnica del CEIC, Pasillo Unidades Administrativas (planta 1 / Peines 6-7), Entrada Por Laboratorios-banco De Sangre Majadahonda, Madrid 28222 Spain  Agencia Española de Medicamentos y Productos Sanitarios (AEMPS) Parque Empresarial Las Mercedes. C/ Campezo, 1 Madrid, Madrid, Communidad de, 28022 Spain | | |
| San Miguel Izquierdo, Jesus, MD (51910) | | CEIC de Navarra Calle Irunlarrea, 3, Recinto Hospital de Navarra, Pabellon de Docencia Pamplona, Navarra, 31008 Spain  CEIC Hospital Puerta de Hierro Majadahonda  C/ Joaquín Rodrigo, 2, Secretaría Técnica del CEIC, Pasillo Unidades Administrativas (planta 1 / Peines 6-7), Entrada Por Laboratorios-banco De Sangre Majadahonda, Madrid 28222 Spain | | |
| De Arriba de La Fuente, Felipe, MD (51902) | | CEIC Hospital Puerta de Hierro Majadahonda  C/ Joaquín Rodrigo, 2, Secretaría Técnica del CEIC, Pasillo Unidades Administrativas (planta 1 / Peines 6-7), Entrada Por Laboratorios-banco De Sangre Majadahonda, Madrid 28222 Spain  CEIC Hospital General Universitario Morales Meseguer Avenida Marques de los Velez s/n Murcia, Murcia, 3008 Spain | | |
| Gonzalez Montes, Yolanda, MD (51904) | | CEIC Hospital Puerta de Hierro Majadahonda  C/ Joaquín Rodrigo, 2, Secretaría Técnica del CEIC, Pasillo Unidades Administrativas (planta 1 / Peines 6-7), Entrada Por Laboratorios-banco De Sangre Majadahonda, Madrid 28222 Spain  CEIC Hospital de Girona Dr Josep Trueta Avenida de Francia, s/n, Planta 9 B Girona, Girona, 17007 Spain | | |
| Mateos Manteca, Maria Victoria, MD (51907) | | CEIC Hospital Puerta de Hierro Majadahonda  C/ Joaquín Rodrigo, 2, Secretaría Técnica del CEIC, Pasillo Unidades Administrativas (planta 1 / Peines 6-7), Entrada Por Laboratorios-banco De Sangre Majadahonda, Madrid 28222 Spain  CEIC Área de Salud de Salamanca Paseo De San Vicente 58-182, Planta 2, Hospital Clínico Universitario - Antiguo Edificio Maternidad Salamanca, Castilla y León, 37007 Spain | | |
| Oriol Rocafiguera, Albert, MD (51908) | | CEIC Hospital Puerta de Hierro Majadahonda  C/ Joaquín Rodrigo, 2, Secretaría Técnica del CEIC, Pasillo Unidades Administrativas (planta 1 / Peines 6-7), Entrada Por Laboratorios-banco De Sangre Majadahonda, Madrid 28222 Spain  CEIC Hospital Universitario Germans Trias i Pujol Carretera Canyet S Badalona, Barcelona, 08916 Spain | | |
| Rosiñol, Laura, MD (51909) | | CEIm Hospital Clinic de Barcelona Calle Villarroel, 170, Agencia de EECC - Servicio de Farmacia, Sotano, Escalera 6B Barcelona, Barcelona, 08036 Spain  CEIC Hospital Puerta de Hierro Majadahonda  C/ Joaquín Rodrigo, 2, Secretaría Técnica del CEIC, Pasillo Unidades Administrativas (planta 1 / Peines 6-7), Entrada Por Laboratorios-banco De Sangre Majadahonda, Madrid 28222 Spain | | |
| Axelsson, Per, MD (52901) Carlson, Kristina, MD (52902) Lund, Johan, MD (52903) Stromberg, Olga, MD | | Regionala Etikprövningsnämnden Lund Sandgatan 1 Lund, Skane lan, 223 50 Sweden | | |
| Gruber, Astrid, MD Hansson, Markus, MD (52904) | | Etikprövningsmyndigheten  von Kraemers allé 4, Segerstedthuset, Box 2110, 750 02 Uppsala Uppsala, Uppsala lan, SE-752 37 Sweden | | |
| Hveding Blimark, Cecilie, MD (52906) | | Regionala Etikprövningsnämnden Lund Sandgatan 1 Lund, Skane lan, 223 50 Sweden  Etikprövningsmyndigheten  von Kraemers allé 4, Segerstedthuset, Box 2110, 750 02 Uppsala Uppsala, Uppsala lan, SE-752 37 Sweden  Regionalt Biobankscentrum Västra Gröna stråket 8 Göteborg, SE-41345 Sweden | | |
| Müller, Rouven, MD (53901) Samaras, Panagiotis, MD | | Kantonale Ethikkommission Zürich Stampfenbachstrasse 121 Zürich, 8090 Switzerland | | |
| Chen, Chih-Cheng, MD (54901) Lin, Doung-Liang (54904) Wang, Po-Nan, MD | | Chang Gung Medical Foundation 199, Tung Hwa North Road Taipei 10507 Taiwan, Province of China   Taiwan Food and Drug Administration No.161-2, Kunyang St., Nangang Dist., Taipei Taiwan, Province of China | | |
| Huang, Shang-Yi, MD (54903) | | Institution Review Board of National Taiwan University Hospital No.1, Changde-de Street, Zhongzheng Dist Taipei 100 Taiwan, Province of China  Taiwan Food and Drug Administration No.161-2, Kunyang St., Nangang Dist., Taipei Taiwan, Province of China | | |
| Hsiao, Hui-Hua, MD (54902) | | Institutional Review Board, Kaohsiung Medical University Chung-Ho Memorial Hospital  No. 100, Tzyou 1st Road  Kaohsiung City 807 Taiwan, Province of China  Taiwan Food and Drug Administration No.161-2, Kunyang St., Nangang Dist., Taipei Taiwan, Province of China | | |
| Beksac, Meral, MD (55901) Goker, Hakan, MD (55904) Unal, Ali, MD (55902) Sonmez, Memet, MD (55908) | | Ankara University Medical Faculty Ethics Committee  Sihhiye, Ankara University School Of Medicine Deanery, Morphology Building, Floor 2 Ankara, Ankara 06100 Turkey | | |
| Korenkova, Sybiryna, MD (56901) Karamanesht, Ievgenii, MD | | Commission on Ethics Questions of Kyiv Center of Bone Marrow Transplantation 119/121 Peremohy Avenue Kyiv 03115 Ukraine | | |
| Chaidos, Aristeidis, MD (57901) | | London - Chelsea Research Ethics Committee Bristol Centre, Level 3, Block B Whitefriars, Lewins Mead Bristol BS1 2NT  United Kingdom  Imperial College Healthcare National Health Service Trust UK R&D Fulham Palace Road, Research and Development, Joint Research Compliance Office, Academic Health Science Centre, Room 5L10, 5th Floor, Lab Block, Charing Cross Hospital London, W6 8RF United Kingdom | | |
| Jenner, Matthew, MBBS (57912) Agis, Hermine, MD | | University Hospital Southampton National Health Service Foundation Trust UK R&D Tremona Road, Research and Development Support Office, SGH, Level E, Laboratory and Pathology Block, Southampton General Hospital  Southampton United Kingdom  London - Chelsea Research Ethics Committee Bristol Centre, Level 3, Block B Whitefriars, Lewins Mead Bristol BS1 2NT  United Kingdom | | |
| Chantry, Andrew, MBCLB (57911) | | Sheffield Teaching Hospitals NHS Foundation Trust UK R&D Whitham Road, Weston Park Hospital Sheffield, York, S10 2SJ United Kingdom  London - Chelsea Research Ethics Committee Bristol Centre, Level 3, Block B Whitefriars, Lewins Mead Bristol BS1 2NT  United Kingdom | | |
| Parrish, Christopher, MD (57910) Cook, Gordon, MBBS | | Leeds Teaching Hospital NHS Foundation Trust Research and Development 34 Hyde Terrace Leeds, LS2 9LN United Kingdom  London - Chelsea Research Ethics Committee Bristol Centre, Level 3, Block B Whitefriars, Lewins Mead Bristol BS1 2NT  United Kingdom | | |
| Ramasamy, Karthik, MBBS (57908) | | Oxford University Hospitals National Health Service Trust UK R&D Old Road, Churchill Hospital Oxford, OX2 7JL United Kingdom  London - Chelsea Research Ethics Committee Bristol Centre, Level 3, Block B Whitefriars, Lewins Mead Bristol BS1 2NT  United Kingdom | | |
| Boyd, Kevin, MD (57907) Kaiser, Martin, MD | | Royal Marsden NHS Foundation Trust R&D Downs Road, The Royal Marsden Hospital NHS Foundation Trust Sutton, SM2 5PT United Kingdom  London - Chelsea Research Ethics Committee Bristol Centre, Level 3, Block B Whitefriars, Lewins Mead Bristol BS1 2NT  United Kingdom  HRA Jarrow BusinnessCentre, Room 001 Jarrow, NE32 3DT United Kingdom | | |
| Garg, Mamta, MD (57906) | | University Hospitals of Leicester NHS Trust Leicester Royal Infirmary Leicester, Leichestershire, LE1 5 United Kingdom  London - Chelsea Research Ethics Committee Bristol Centre, Level 3, Block B Whitefriars, Lewins Mead Bristol BS1 2NT  United Kingdom | | |
| Wechalekar, Ashutosh, MBBS (57905) | | University College London Hospitals (UCLH) - R&D 149 Tottenham Court Road, 1st floor, Maple House London, W1P 9LL United Kingdom  Royal Free London NHS Foundation Trust R&D Pond Street, UCL Medical School, Royal Free Campus, Ground Floor, Room 649 London, NW3 2QG United Kingdom  London - Chelsea Research Ethics Committee Bristol Centre, Level 3, Block B Whitefriars, Lewins Mead Bristol BS1 2NT  United Kingdom | | |
| Sati, Hamdi, MD (57903) Mohite, Unmesh | | London - Chelsea Research Ethics Committee Bristol Centre, Level 3, Block B Whitefriars, Lewins Mead Bristol BS1 2NT  United Kingdom  Abertawe Bro Morgannwg University Health Board UK R&D One Talbot Gateway, Baglan Energy Park Port Talbot, SA12 7BR United Kingdom | | |
| Oakervee, Heather, BSc (57902) Hallam, Simon, MD | | London - Chelsea Research Ethics Committee Bristol Centre, Level 3, Block B Whitefriars, Lewins Mead Bristol BS1 2NT  United Kingdom  Barts Health National Health Service Trust R&D 5 Walden Street, Research and Development, The Joint Research Management Office, Queen Mary's Innovation Centre, Lower Ground Floor London, E1 2EF United Kingdom | | |
| Benjamin, Reuben (57904) Schey, Stephen, MBBS | | Guy's and St Thomas' NHS Foundation Trust R&D Westminster Bridge Road London, London, City of, SE1 7EH United Kingdom  London - Chelsea Research Ethics Committee Bristol Centre, Level 3, Block B Whitefriars, Lewins Mead Bristol BS1 2NT  United Kingdom | | |
| Verma, Amit, MD (58907) Battini, Ramakrishna, MD Janakiram, Murali, MBBS TBC | | Biomedical Research Alliance of New York, LLC/ Institutional Review Board 1981 Marcus Avenue, Suite 210 Lake Success, New York 11042 United States | | |
| Berryman, Robert, MD (58906) | | Baylor Research Institute 3310 Live Oak, Suite 500 Dallas, Texas, 75204 United States  Baylor Scott and White Research Institute 2401 S 31st St Temple, Texas, 76508-0001 United States  Baylor College of Medicine IRB 1 Baylor Plz Rm 713D Houston, Texas, 77030-3411 United States | | |
| Buadi, FrancisMD (58905) Dingli, David, MD | | Mayo Clinic Institutional Review Board 200 1st St SW Rm 4-60Rochester, MN 55905-0001 United States | | |
| Akashi, Koichi, MD (63910) | | Kyushu University Hospital IRB 3-1-1, Notame, Minami-Ku Fukuoka, Fukuoka 812-8582 Japan | | |
| Ishiguro, Takuro, MD (63909) Chou, Takaaki, MD | | Niigata Cancer Center Hospital IRB 2-15-3, Kawagishi-cho, Chuo-ku Niigata-shi, Niigata 951-8133 Japan | | |
| Sekine, Rieko, MD (63913) Togano, Tomiteru (Former PI) Hagiwara, Shotaro, MD (Former PI) | | National Center for Global Health and Medicine Hospital IRB1-21-1 Toyama Shinjuku-ku, Tokyo 162-8655 Japan | | |
| Iida, Shinsuke, MD (63902) | | Nagoya City University Hospital Institutional Review Board 1-Kawasumi, Mizuho-cho, Mizuho-ku Nagoya-City, 467-8602 Japan  Nagoya City Hospital Institutional Review Board 1-2-23 Wakamizu,Chikusa-ku Nagoya, Aiti, 464-8547 Japan  Kyushu University Hospital IRB 3-1-1 Maidashi, Higashi-ku Fukuoka-City, 812-8582 Japan | | |
| Ishikawa, Takayuki, MD (63904) | | Kobe City Medical Center General Hospital IRB 2-1-1, Minatojima-minamimachi, Chuo-ku Kobe-shi, Hyogo 650-0047 Japan | | |
| Saito, Akio, MD (63905) Matsumoto, Morio, MD | | NHO Nishigunma National Hospital  2854, Kanai Shibukawa, Gunma 377-8511 Japan  NHO Shibukawa Medical Center 383, Shiroi Shibukawa-city, Gunma 377-0204 Japan | | |
| Nagai, Hirokazu, MD (63906) | | NHO Nagoya Medical Center Instiituitional Review Board  4-1-1, Sannomaru, Naka-ku Nagoya, Aichi 460-0001 Japan  NHO Shibukawa Medical Center 383 Shiroi, Shibukawa-Shi, Gunma, 377-0204 Japan  Center Hospital of the National Center for Global Health and Medicine Institutional Review Board 1-21-1 Toyama Shinjuku, Tokyo, 162-8655 Japan | | |
| Sakaida, Emiko, MD (63903) Nakaseko, Chiaki, MD | | Chiba University Hospital IRB 1-8-1 lnohana Chuo-ku Chiba, Tokyo 2608677 Japan  Chiba Cancer Center IRB 666-2 Nitona-cho, Chuo-ku Chiba, 260-8717 Japan  NHO Nagoya Medical Center Instiituitional Review Board 4-1-1, Sannomaru, Naka-ku Nagoya, 460-0001 Japan  Chiba Children's Hospital Institutional Review Board 579-1 Hetacho, Midori-Ku Chiba-Shi, Tiba, 266-0007 Japan | | |
| Sunami, Kazutaka, MD (63908) | | National Hospital Organization Okayama Medical Center IRB 1711-1 Tamasu, Kita-ku Okayama, Okayama 701-1154 Japan  Okayama City General Medical Center Okayama City Hospital Institutional Review Board 3-20-1 Kitanagaseomotemachi Kita-ku Okayama, 700-8557 Japan | | |
| Tsukada, Nobuhiro, MD (63906) Suzuki, Kenshi, MD | | Japanese Red Cross Medical Center IRB 4-1-22 Hiroo Shibuya-ku, Tokyo 150-8935 Japan | | |
| Takezako, Naoki, MD (63911) | | National Hospital Organization Disaster Medical Center IRB 3256, Midori-cho Tachikawa, Tokyo 190-0014 Japan | | |
| Na Nakorn, Thanyaphong, MD (64901) | | The Institutional Review Board of the Faculty of Medicine, Chulalongkorn University 1873 Rama 4 Road Bangkok, Krung Thep Maha Nakhon, 10330 Thailand  Drug Control Division of the Food and Drug Administration 88/24 Tiwanond Road, Ministry Of Public Health Nonthaburi, Nonthaburi, 11000 Thailand | | |
| Prayongratana, Kannadit, MD (64902) | | Institutional Review Board, Royal Thai Army Medical Department Office 317, Rajavithi Road, Ratchatavee District, 5th floor, Phramongkutklao Building Bangkok, Krung Thep Maha Nakhon-Bangkok 10400 Thailand  Medical Device Control Division, Thailand Food and Drug Administration, Ministry of Public Health Thiwanon Road Nonthaburi, 11000 Thailand  Drug Control Division of the Food and Drug Administration 88/24 Tiwanond Road, Ministry Of Public Health Nonthaburi, Nonthaburi, 11000 Thailand | | |
