# Supplementary material for: Final OS analyses from the TOURMALINE- MM3 and -MM4 RCTs of ixazomib maintenance in newly diagnosed multiple myeloma
Source: Blood Cancer J. 2025 Dec 4;16(1):15. doi: 10.1038/s41408-025-01411-9 (PMC12808303; doi:10.1038/s41408-025-01411-9)
Supplement: Supplementary file 3 — TOURMALINE-MM4 IRB and IEC Information [file 41408_2025_1411_MOESM3_ESM.doc]

#### IRB and IEC Information

The following table includes a list of IRBs and IECs used by investigators who received study drug, as well as any investigators who replaced them during the study.

| Principal Investigator | IRBs or IECs |
| --- | --- |
| **Bar, Daniel Oscar, MD (01102)** | Comité Independiente de Etica en investigación clínica Dr.  Carlos A. Barclay  Paraná 755, 6º A y B  Ciudad Autónoma de Buenos Aires, C1107  Argentina  Comité Provincial de Bioética - Ministerio de Salud de la Provincia de Santa Fé  Blvd Gálvez 1563  Santa Fé, Santa Fe, 3000  Argentina |
| **Fantl, Dorotea Beatriz Eugenia, MD (01105)** | Comité de Ética de Protocolos de Investigación del Hospital Italiano (CEPI) Tte. General Juan Domingo Perón 4190 CABA, Buenos Aires C1181ACH Argentina |
| **Jarchum, Gustavo Daniel, MD (01103)** | Comité Institucional de Ética de Investigación en Salud del Sanatorio Allende  Av Hipólito Yrigoyen 384  Cordoba, Cordoba X5000JHGQ  Argentina  Consejo de Evaluación Ética de Investigación en Salud – CoEIS  Avenida Velez Sarsfield 2311  Córdoba, Córdoba, 5000  Argentina |
| **Riveros, Dardo Alberto, MD (01101)** | Comité de Ética en Investigación de Cemic Galván 4102  CABA, Buenos Aires C1431FWO  Argentina |

| Principal Investigator | IRBs or IECs |
| --- | --- |
| **Quach, Hang, Dr (02103)** | Melbourne Health Human Research Ethics Committee  Grattan Street, Human Research Ethics Committee Directorate, Post Office  Parkville, Victoria, 3050  Australia  Monash Health Human Research Ethics Committee A  Monash Medical Centre  246 Clayton Road  Clayton, Victoria 3004  Australia  Southern Health Human Research Ethics Committee A  246 Clayton Road  Clayton, Victoria 3004  Australia  St Vincent's Hospital Melbourne Human Research Ethics Committee  27 Victoria Parade  Fitzroy, Victoria 3065  Australia |
| **Spencer, Andrew, Prof (02101)** | Monash Health Human Research Ethics Committee A  Monash Medical Centre  246 Clayton Road  Clayton, Victoria 3004  Australia  Southern Health Human Research Ethics Committee A  246 Clayton Road  Clayton, Victoria 3004  Australia |
| **Walker, Patricia Ann, Dr (02102)**  **Catalano, John, A/Prof (Former PI)** | Melbourne Health Human Research Ethics Committee  Grattan Street, Human Research Ethics Committee Directorate, Post Office  Parkville, Victoria, 3050  Australia  Monash Health Human Research Ethics Committee A  Monash Medical Centre  246 Clayton Road  Clayton, Victoria 3004  Australia  Peninsula Health Human Research Ethics Committee  Hastings Road  Frankston, Victoria, 3199  Australia  Southern Health Human Research Ethics Committee A  246 Clayton Road  Clayton, Victoria 3004  Australia |
| **Agis, Hermine, Priv. Doz. Dr (03104)** | Ethikkommission der Medizinischen Universität Wien  Borschkegasse 8b  Wien 1090  Austria  Ethikkommission für das Bundesland Salzburg  Michael-Pacher-Straße  Salzburg 5020  Austria |
| **Greil, Richard, Univ. Prof. Dr (03102)** | Ethikkommission für das Bundesland Salzburg  Michael-Pacher-Straße  Salzburg 5020  Austria |
| **Gunsilius, Eberhard, Univ. Doz. Dr. med**  **(03105)** | Ethikkommission der Medizinischen Universität Innsbruck  Innrain 43, Innsbruck  Tirol 6020  Austria  Ethikkommission für das Bundesland Salzburg  Michael-Pacher-Straße  Salzburg 5020  Austria |
| **Kühr, Thomas, Univ. Doz. Dr (03101)** | Ethikkommission des Landes Oberösterreich  Krankenhausstraße 5, Linz  Oberösterreich 4020  Austria  Ethikkommission für das Bundesland Salzburg  Michael-Pacher-Straße  Salzburg 5020  Austria |
| **Vekemans, Marie-Christiane (04102)** | Comité d’Ethique hospitalo-facultaire Cliniques universitaires Saint-Luc  Promenade de l’Alma 51 bte B1.43.03  Brussels 1200  Belgium  Commissie Medische Ethiek UZ Brussel  Laarbeeklaan 101  Brussels 1090  Belgium  EC UZ Brussel  Laarbeeklaan 101  Brussels 1090  Belgium |
| **Bellesso, Marcelo, MD (05102)**  **Chialanza Garcia, Laura Yolanda, MD (Former PI)** | Comissão Nacional de Ética em Pesquisa – CONEP  SRTV 701, Via W 5 Norte, lote D  Edifício PO 700, 3° andar, Asa Norte  Brasília, DF 70719-040  Brazil  Comitê de Ética em Pesquisa da faculdade de Medicina do ABC  Av Lauro Gomes, 2000  Vila Sacadura Cabral  Santo André, São Paulo 09060-870  Brazil |
| **Bonito, Debora Rodrigues, MD (05107)**  **Cubero, Daniel de Iracema Gomes, MD (Former PI)** | Comissão Nacional de Ética em Pesquisa – CONEP  SRTV 701, Via W 5 Norte, lote D  Edifício PO 700, 3° andar, Asa Norte  Brasília, DF 70719-040  Brazil  Comitê de Ética em Pesquisa da faculdade de Medicina do ABC  Avenida Principe De Gales 821  Santo André, São Paulo 09060-870  Brazil |
| **Capra, Marcelo Eduardo Zanella, MD (05101)** | Comissão Nacional de Ética em Pesquisa – CONEP  SRTV 701, Via W 5 Norte, lote D  Edifício PO 700, 3° andar, Asa Norte  Brasília, DF 70719-040  Brazil  Comitê de Ética em Pesquisa - Hospital Mãe de Deus  Rua José de Alencar, 286  Menino Deus  Porto Alegre, RS 90880-480  Brazil |
| **Crusoé, Edvan de Queiroz, MD (05115)** | Comissão Nacional de Ética em Pesquisa – CONEP  SRTV 701, Via W 5 Norte, lote D  Edifício PO 700, 3° andar, Asa Norte  Brasília, DF 70719-040  Brazil  Comitê de Ética em Pesquisa em Seres Humanos do Centro de Pesquisas Gonçalo Moniz – FIOCRUZ Bahia – BA  Rua Waldemar Falcão, 121  Candeal  Salvador, BA 40296-710  Brazil |
| **de Mattos, Ederson Roberto, MD (05123)** | Comissão Nacional de Ética em Pesquisa – CONEP  SRTV 701, Via W 5 Norte, lote D  Edifício PO 700, 3° andar, Asa Norte  Brasília, DF 70719-040  Brazil  Comitê de Ética em Pesquisa da Fundação Hospital Amaral  Carvalho  Rua das Palmeiras, 89  Vila Assis  Jaú, SP 17210-120  Brazil |
| **Duarte, Gislaine Oliveira, MD (05122)** | Comissão Nacional de Ética em Pesquisa – CONEP  SRTV 701, Via W 5 Norte, lote D  Edifício PO 700, 3° andar, Asa Norte  Brasília, DF 70719-040  Brazil  Comitê de Ética em Pesquisa da Faculdade de Ciencias Medicas - UNICAMP/SP  Rua Tessalia Vieira de Camargo, 126  Campinas, SP 13083-970  Brazil |
| **Franke, Fábio André, MD (05119)** | Comissão Nacional de Ética em Pesquisa – CONEP  SRTV 701, Via W 5 Norte, lote D  Edifício PO 700, 3° andar, Asa Norte  Brasília, DF 70719-040  Brazil  Comitê de Ética em Pesquisa da Universidade Regional do Noroeste do Estado do Rio Grande do Sul – Un  Rua do Comércio, 3.000 - Subsolo do Prédio da Biblioteca – Sala BIB – S 06. Bairro Universitário.  Ijuí 98700-000  Brazil |
| **Gastal, Gabriela Roncone, MD (05120)** | Comissão Nacional de Ética em Pesquisa – CONEP  SRTV 701, Via W 5 Norte, lote D  Edifício PO 700, 3° andar, Asa Norte  Brasília, DF 70719-040  Brazil  Comitê de Ética em Pesquisa em Seres Humanos do Hospital Municipal São José  Rua Plácido Gomes, 488  Anita Garibaldi  Joinville, SC 89202-050  Brazil |
| **Gonçalves, Iara Zapparoli, MD (05113)** | Comissão Nacional de Ética em Pesquisa – CONEP  SRTV 701, Via W 5 Norte, lote D  Edifício PO 700, 3° andar, Asa Norte  Brasília, DF 70719-040  Brazil  Comitê de Ética em Pesquisa Fundação Pio XII Hospital de Câncer de Barretos  Rua Antenor Duarte Vilela, 1331  Barretos, SP 14784-400  Brazil |
| **Guimarães, Antônio Júlio de Ulyssea, MD (05125)** | Comissão Nacional de Ética em Pesquisa – CONEP  SRTV 701, Via W 5 Norte, lote D  Edifício PO 700, 3° andar, Asa Norte  Brasília, DF 70719-040  Brazil  Comitê de Ética em Pesquisa do Instituto Estadual de Hematologia Arthur de Siqueira Cavalcanti  Rua Frei Caneca, 8 Centro  Rio de Janeiro, RJ 20211-030  Brazil |
| **Hamerschlak, Nelson, MD (05108)** | Comissão Nacional de Ética em Pesquisa – CONEP  SRTV 701, Via W 5 Norte, lote D  Edifício PO 700, 3° andar, Asa Norte  Brasília, DF 70719-040  Brazil  Comite de Etica em Pesquisa em Seres Humanos do Hospital Israelita Albert Einstein  Av Albert Einstein, 627  São Paulo, 05652-000  Brazil |
| **Hungria, Vânia Tietsche de Moraes, MD (05126)** | Comissão Nacional de Ética em Pesquisa – CONEP  SRTV 701, Via W 5 Norte, lote D  Edifício PO 700, 3° andar, Asa Norte  Brasília, DF 70719-040  Brazil  Comite de Etica em Pesquisa da Universidade Federal de Sao Paulo - Hospital Sao Paulo  Rua Botucatu 572  São Paulo, São Paulo  04023-062  Brazil  Comitê de Ética em Pesquisa em Seres Humanos da Santa Casa de São Paulo  Rua Santa Isabel, 305 - Santa Cecília  São Paulo, São Paulo  01221-0100  Brazil |
| **Maiolino, Angelo, MD (05124)** | Comissão Nacional de Ética em Pesquisa – CONEP  SRTV 701, Via W 5 Norte, lote D  Edifício PO 700, 3° andar, Asa Norte  Brasília, DF 70719-040  Brazil  Comite de Etica em Pesquisa do Hospital Universitario Clementino Fraga Filho/UFRJ  Rua Professor Rodolpho Paulo Rocco 255  Rio De Janeiro, Rio De Janeiro  21941-913  Brazil |
| **Miguel, Carlos Eduardo, MD (05110)** | Comissão Nacional de Ética em Pesquisa – CONEP  SRTV 701, Via W 5 Norte, lote D  Edifício PO 700, 3° andar, Asa Norte  Brasília, DF 70719-040  Brazil  Comitê de Ética em Pesquisa em Seres Humanos Faculdade de Medicina de São José do Rio Preto  Avenida Brigadeiro Faria Lima 5416, Vila São Pedro  São José Do Rio Preto  15090-000  Brazil |
| **Munhoz, Eduardo Cilião (05111)** | Comissão Nacional de Ética em Pesquisa – CONEP  SRTV 701, Via W 5 Norte, lote D  Edifício PO 700, 3° andar, Asa Norte  Brasília, DF 70719-040  Brazil  Comite de Etica em Pesquisa em Seres Humanos Liga Paranaense de Combate ao Cancer  Rua 239 Numbero 206  Goiás, Goiás  74605-070  Brazil |
| **Palladino, Alexandre de Mendonca, MD (05112)**  **Scheliga, Adriana Alves de Souza, MD (Former PI)** | Comissão Nacional de Ética em Pesquisa – CONEP  SRTV 701, Via W 5 Norte, lote D  Edifício PO 700, 3° andar, Asa Norte  Brasília, DF 70719-040  Brazil  Comitê de Ética em Pesquisa em Seres Humanos do Instituto Nacional do Câncer (INCA)  Rua do Resende 128  Rio de Janeiro, Rio de Janeiro  20231-092  Brazil |
| **Rabelo, Yana de Sousa, MD (05109)**  **Cordeiro de Farias, Danielle Leão, MD (Former PI)** | Comissão Nacional de Ética em Pesquisa – CONEP  SRTV 701, Via W 5 Norte, lote D  Edifício PO 700, 3° andar, Asa Norte  Brasília, DF 70719-040  Brazil  Comite de Etica em Pesquisa em Seres Humanos Hospital das Clinicas da Universidade Federal de Goias  1º Avenida S/N  Goiania, Goiás  74605-050  Brazil |
| **Rodrigues de Oliveira, José Salvador, MD (05116)** | Comissão Nacional de Ética em Pesquisa – CONEP  SRTV 701, Via W 5 Norte, lote D  Edifício PO 700, 3° andar, Asa Norte  Brasília, DF 70719-040  Brazil  Comite de Etica em Pesquisa – CEP da Casa de Saude Santa Marcelina  Rua Santa Marcelina, 177  São Paulo, São Paulo  062270-070  Brazil |
| **Schaan, Mariza D’Agord, MD (05128)** | Comissão Nacional de Ética em Pesquisa – CONEP  SRTV 701, Via W 5 Norte, lote D  Edifício PO 700, 3° andar, Asa Norte  Brasília, DF 70719-040  Brazil  Comite de Etica em Pesquisa da Pontificia Universidade Catolica do Rio Grande do Sul - PUC/RS  Avenida Ipiranga, 6681  Porto Alegre  Rio Grande do Sul, RS 90619-900  Brazil |
| **Schusterschitz da Silva Araujo, Sérgio, MD (05103)** | Comissão Nacional de Ética em Pesquisa – CONEP  SRTV 701, Via W 5 Norte, lote D  Edifício PO 700, 3° andar, Asa Norte  Brasília, DF 70719-040  Brazil  Comitê de Ética em Pesquisa em Seres Humanos da Universidade Federal de Minas Gerais  Av. Antônio Carlos, 6627 - Campus Pampulha  Belo Horizonte  Minas Gerais, 31270-901  Brazil |
| **Vieira, Garles Miller Matias, MD (05117)** | Comissão Nacional de Ética em Pesquisa – CONEP  SRTV 701, Via W 5 Norte, lote D  Edifício PO 700, 3° andar, Asa Norte  Brasília, DF 70719-040  Brazil  Comitê de Ética em Pesquisa da Fundação Antônio Prudente - AC Camargo Câncer Center  Rua Professor Antonio Prudente 211  São Paulo, São Paulo  01509-900  Brazil |
| **Villarim, Carolina Colaço, MD (05104)**  **Maciel, James Farley Rafael, MD (Former PI)** | Comissão Nacional de Ética em Pesquisa – CONEP  SRTV 701, Via W 5 Norte, lote D  Edifício PO 700, 3° andar, Asa Norte  Brasília, DF 70719-040  Brazil  Comitê de Ética em Pesquisa em Seres Humanos da Liga Norte Riograndense Contra o Câncer  Rua Dr. Mário Negócio, 2267  Natal  Rio Grande do Norte, 59040-000  Brazil |
| **Weber, Cristiane Seganfredo, MD (05118)** | Comissão Nacional de Ética em Pesquisa – CONEP  SRTV 701, Via W 5 Norte, lote D  Edifício PO 700, 3° andar, Asa Norte  Brasília, DF 70719-040  Brazil  Comitê de Ética em Pesquisa do Hospital de Clínicas de Porto Alegre  Rua Ramiro Barcelos, 2350 Sala 2227 2º Andar  Porto Alegre  Rio Grande do Sul, 90035-003  Brazil |
| **Kukreti, Vishal, Dr (7101)** | University Health Network Research Ethics Board  17th Floor Suite  Toronto, ON M5G 1Z6  Canada |
| **Kuruvilla, Philip, Dr (7103)**  **Sehdev, Sandeep, Dr (Former PI)** | William Osler Health System Research Ethics Board  2100 Bovaird Drv. East  Brampton, ON, L6R 3J7  Canada |
| **Shustik, Chaim, MD, Dr (7102)** | McGill University Health Center Montreal Hospital  03  Montreal, QC, H3H 2R9  Canada |
| **Rojas Hopkins, Christine Michelle, MD (8103)** | Comite de Etica Cientifico del Servicio de Salud Metropolitano Oriente Pediatrico  Avenida Antonio Varas 360  Santiago  Chile  Comite Etico Cientifico Clinica Reñaca  Anabaena 336  Vina del Mar, 2540364  Chile  Comité Ético Científico del Servicio de Salud Viña del Mar Quillota-Hospital Gustavo Fricke  Calle Álvarez 1532  Viña del Mar  Valparaíso, 2520000  Chile |
| **Yañez Ruiz, Eduardo Patricio, MD (8102)** | Comité de Evaluación Ética Cientifica del Servicio de Salud  Araucanía Sur  Andrés Bello 636  Temuco, Araucania, 4791301  Chile |
| **Chen, Lijuan (9114)** | Jiangsu Province Hospital Ethics Committee  300 Guangzhou Road  Nanjing City, Jiangsu, 210029  China  Jiangsu Province Hospital GCP Office  Building No. 300, Guangzhou Rd.  Nanjing City, Jiangsu, 210029  China |
| **Chen, Wenming (9105)** | EC of Beijing Chaoyang Hospital Capital Medical University  No. 8 Gongti South Rd., Chaoyang District  Beijing, Beijing 100043  China |
| **Du, Juan (09104)**  **Fu, Weijun (Former PI)** | Ethical Committee of Shanghai Changzheng Hospital  415 Fengyang Rd.  Huangpu District  Shanghai, 200003  China |
| **Li, Jian (9107)** | Ethics Committee of Peking Union Medical College Hospital  No.1 Shuai Fu Yuan, Dongcheng District  Beijing, Beijing, 100730  China |
| **Liu, Yan (9106)** | EC of Peking University Third Hospital  No. 49, Garden North Rd. Haidian District  Beijing, Beijing, 100191  China |
| **Meng, Haitao (9101)** | EC of The First Affiliated Hospital, Zhejiang University School of Medicine  No.58 Chengzhan Road  Hangzhou Zehjiang 310009  China |
| **Galvez Cardenas, Kenny Mauricio, MD (10101)** | Comite de Investigaciones y Etica en Investigaciones del Hospital Pablo Tobon Uribe  Calle 78B No.69-240  Medellin, Antioquia  050034  Colombia  Comite de Etica e Investigacion Instituto Nacional de Cancerologia  Calle 1 No. 9-85  Bogotá, Cundinamarca, 110311  Colombia |
| **Herrera Jauregui, María Victoria, MD (10104)** | Comité de Investigaciones y Ética Institucional (CIEI)  Carrera 7 No. 40-62  Bogota, Cundinamarca, 110231  Colombia |
| **Pereira Garzón, Alberto Mario (10103)**  **Bermúdez Silva, Carlos Daniel (Former PI)** | Comité de Ética e Investigaciones del Instituto Nacional de Cancerología  Calle 1 No. 9-85  Bogotá, Cundinamarca, 110311  Colombia |
| **Hájek, Roman, CSc, Prof., MUDr. (12105)** | Etická komise Fakultní nemocnice Královské Vinohrady  Šrobárova 1150/50  Praha – 10, 10034  Czech Republic  Etická komise Fakultní nemocnice Ostrava  17. listopadu 1790  Ostrava – Moravskoslezský kraj, 708 52  Czech Republic |
| **Maisnar, Vladimír, CSc, Prof., MUDr.**  **(12106)** | Etická komise Fakultní nemocnice Královské Vinohrady  Šrobárova 1150/50  Praha – 10, 10034  Czech Republic  Eticka komise Fakultni nemocnice Hradec Kralove  Sokolská 581  Hradec Králové, Královéhradecký kraj, 500 12  Czech Republic |
| **Pavlicek, Petr, MUDr. (12102)**  **Gregora, Evzen, MUDr. (Former PI)** | Etická komise Fakultní nemocnice Královské Vinohrady  Šrobárova 1150/50  Praha – 10, 10034  Czech Republic |
| **Pika, Tomas, (12104)**  **Scudla, Vlastimil, MUDr., CSc, Prof. (Former PI)** | Etická komise Fakultní nemocnice Královské Vinohrady  Šrobárova 1150/50  Praha – 10, 10034  Czech Republic  Eticka komise Fakultni nemocnice Olomouc  I. P. Pavlova 185/6  Olomouc, Olomoucký kraj, 779 00  Czech Republic |
| **Pour, Ludek, PhD, MUDr. (12101)** | Etická komise Fakultní nemocnice Královské Vinohrady  Šrobárova 1150/50  Praha – 10, 10034  Czech Republic  Eticka komise Fakultni nemocnice Brno  Jihlavská 340/20  Brno, Jihomoravský kraj, 625 00  Czech Republic |
| **Špicka, Ivan, CSc., MUDr., Prof. (12103)** | Etická komise Fakultní nemocnice Královské Vinohrady  Šrobárova 1150/50  Praha – 10, 10034  Czech Republic  Eticka komise Vseobecne fakultni nemocnice v Praze  Na Bojišti 1771/1  Praha, Praha, hlavní mesto, 120 00  Czech Republic |
| **Abildgaard, Niels (13102)** | De Videnskabsetiske Komitéer for Region Syddanmark  Damhaven 12  Vejle 7100  Denmark  De Videnskabsetiske Komiteer i Region Hovedstaden  Blegdamsvej 60  København Ø  Capital, 2100  Denmark |
| **Andersen, Niels Frost (13103)** | De Videnskabsetiske Komitéer for Region Syddanmark  Damhaven 12  Vejle 7100  Denmark  De Videnskabsetiske Komiteer i Region Hovedstaden  Blegdamsvej 60  København Ø  Capital, 2100  Denmark |
| **Helleberg, Carsten (13104)** | De Videnskabsetiske Komitéer for Region Syddanmark  Damhaven 12  Vejle 7100  Denmark  De Videnskabsetiske Komiteer i Region Hovedstaden  Blegdamsvej 60  København Ø  Capital, 2100  Denmark |
| **Pedersen, Robert Schou (13101)** | De Videnskabsetiske Komitéer for Region Syddanmark  Damhaven 12  Vejle 7100  Denmark  De Videnskabsetiske Komiteer i Region Hovedstaden  Blegdamsvej 60  København Ø  Capital, 2100  Denmark |
| **Choquet, Sylvain (18110)** | Comité de Protection des Personnes Nord-Ouest IV  6 rue du Professeur Laguesse – CS70 001  Bâtiment ex USN B (RDC)  Lille Cedex 59037  France |
| **Facon, Thierry (18103)** | Comité de Protection des Personnes Nord-Ouest IV  6 rue du Professeur Laguesse – CS70 001  Bâtiment ex USN B (RDC)  Lille Cedex 59037  France |
| **Feugier, Pierre (18109)**  **Clement-Filliatre, Lauriane (Former PI)**  **Perrot, Aurore (Former PI)** | Comité de Protection des Personnes Nord-Ouest IV  6 rue du Professeur Laguesse – CS70 001  Bâtiment ex USN B (RDC)  Lille Cedex 59037  France |
| **Frenzel, Laurent (18112)** | Comité de Protection des Personnes Nord-Ouest IV  6 rue du Professeur Laguesse – CS70 001  Bâtiment ex USN B (RDC)  Lille Cedex 59037  France |
| **Leleu, Xavier (18105)** | Comité de Protection des Personnes Nord-Ouest IV  6 rue du Professeur Laguesse – CS70 001  Bâtiment ex USN B (RDC)  Lille Cedex 59037  France |
| **Moreau, Philippe (18113)** | Comité de Protection des Personnes Nord-Ouest IV  6 rue du Professeur Laguesse – CS70 001  Bâtiment ex USN B (RDC)  Lille Cedex 59037  France |
| **Blau, Igor, Dr. med. (19104)** | Ethikkommission der Universität Ulm  Helmholtzstraße 20  Ulm 89081  Germany |
| **Burkart, Christof, Dr. (19108)**  **Brugger, Wolfram, Prof., Dr., med. (Former PI)** | Ethikkommission der Universität Ulm  Helmholtzstraße 20  Ulm 89081  Germany |
| **Dürig, Jan, Prof., Dr. med. (19101)** | Ethik-Kommission der Medizinischen Fakultät der Universität Duisburg-Essen  Robert-Koch-Straße 9-11  Essen, Nordrhein-Westfalen  45147  Germany  Ethikkommission der Universität Ulm  Helmholtzstraße 20  Ulm 89081  Germany |
| **Hubmann, Max, Dr. (19112)** | Ethikkommission der Universität Ulm  Helmholtzstraße 20  Ulm 89081  Germany |
| **Kaddu-Mulindwa, Dominic, Dr. (19105)**  **Adrian, Nicole, Dr., med. (Former PI)**  **Pfreundschuh, Michael, Prof., Dr., med. (Former PI)** | Ethik-Kommission bei der Ärztekammer des Saarlandes  Faktoreistrasse 4  Saarbrücken  Saarland, 66111  Germany  Ethikkommission der Universität Ulm  Helmholtzstraße 20  Ulm 89081  Germany |
| **Kull, Miriam, Dr. (19106)**  **Langer, Christian, PD, Dr. (Former PI)** | Ethikkommission der Universität Ulm  Helmholtzstraße 20  Ulm 89081  Germany |
| **Munder, Markus, Prof., Dr. (19110)** | Ethikkommission der Universität Ulm  Helmholtzstraße 20  Ulm 89081  Germany |
| **Schmidt, Christian, Dr. (19109)**  **Ostermann, Helmut, Dr., med. (Former PI)** | Ethikkommission der Universität Ulm  Helmholtzstraße 20  Ulm 89081  Germany |
| **Schöttker, Björn (19119)**  **Schlag, Rudolf, Dr. (Former PI)** | Ethikkommission der Universität Ulm  Helmholtzstraße 20  Ulm 89081  Germany |
| **Delimpasi, Sosana (20104)** | National Ethics Committee (EED)  284 Mesogeion Avenue  Athens, Attiki 15562  Greece |
| **Dimopoulos, Athanasios-Meletios (20103)** | National Ethics Committee (EED)  284 Mesogeion Avenue  Athens, Attiki 15562  Greece |
| **Iskas, Michalis (20101)**  **Anagnostopoulos, Achilles (Former PI)** | National Ethics Committee (EED)  284 Mesogeion Avenue  Athens, Attiki 15562  Greece |
| **Kapsali, Eleni (20102)**  **Briasoulis, Evangelos (Former PI)** | National Ethics Committee (EED)  284 Mesogeion Avenue  Athens, Attiki 15562  Greece |
| **Katodritou, Eirini (20106)** | National Ethics Committee (EED)  284 Mesogeion Avenue  Athens, Attiki 15562  Greece |
| **Vassilopoulos, George (20105)** | National Ethics Committee (EED)  284 Mesogeion Avenue  Athens, Attiki 15562  Greece |
| **Borbényi, Zita, Dr., PhD (22104)** | Egeszsegugyi Tudomanyos Tanacs Klinikai Farmakologiai Etikai Bizottsag  Arany János utca 6-8.  Budapest, 1051  Hungary  Egeszsegugyi Tudomanyos Tanacs Tudomanyos es Kutatasetikai Bizottsaga  Szechenyi Istvan ter 7-8  Budapest, 1051  Hungary |
| **Illés, Árpád, Prof., Dr. (22102)** | Egeszsegugyi Tudomanyos Tanacs Klinikai Farmakologiai Etikai Bizottsag  Arany János utca 6-8.  Budapest, 1051  Hungary  Egeszsegugyi Tudomanyos Tanacs Tudomanyos es Kutatasetikai Bizottsaga  Szechenyi Istvan ter 7-8  Budapest, 1051  Hungary |
| **Mikala, Gabor, Dr. (22105)** | Egeszsegugyi Tudomanyos Tanacs Klinikai Farmakologiai Etikai Bizottsag  Arany János utca 6-8.  Budapest, 1051  Hungary |
| **Nagy, Zsolt, PhD, Dr. (22103)** | Egeszsegugyi Tudomanyos Tanacs Klinikai Farmakologiai Etikai Bizottsag  Arany János utca 6-8.  Budapest, 1051  Hungary  Egeszsegugyi Tudomanyos Tanacs Tudomanyos es Kutatasetikai Bizottsaga  Szechenyi Istvan ter 7-8  Budapest, 1051  Hungary |
| **Ben-Yehuda, Dina, Prof. (27105)** | Institutional Helsinki Committee  Hadassah University Hospital EC  Kiryat Hadassab,  P.O. Box 12000  Jerusalem, 91120  Israel |
| **Cohen, Yael, Dr. (27103)** | Institutional Helsinki Committee  Tel-Aviv Sourasky Medical Center EC  6 Weitzmann St.  Tel-Aviv, 64239  Israel |
| **Gutwein, Odit, Dr. (27106)**  **Kornberg, Abraham, Dr. (Former PI)** | Shamir Medical Center Assaf Harofeh EC  Beer Yaakov  Zerifin,  70300  Israel |
| **Itchaki, Gilad, Dr. (27101)**  **Vaxman, Iuliana, Dr. (Former PI)**  **Magen-Nativ, Hila, Dr. (Former PI)** | Institutional Helsinki Committee  Rabin Medical Center EC  39 Jabotinsky St.  Petach Tikva, 49100  Israel |
| **Jarchowsky Dolberg, Osnat, Dr. (27102)**  **Hardan, Izhar, Dr. (Former PI)** | Institutional Helsinki Committee  Meir Medical Center EC  59 Tchernichovsky St.  Kfar, Saba 44281  Israel |
| **Nagler, Arnon, Prof. (27108)** | Institutional Helsinki Committee  Chaim Sheba Medical Center EC  2 Sheba Rd., Tel-Hashomer  Ramat-Gan,  52621  Israel |
| **Shpilberg, Ofer, Prof. (27110)** | Assuta Hospital Systems  20 Habarzel Street  Tel-Aviv-Yafo  Tel-Aviv , 60000  Israel |
| **Tadmor, Tamar, Dr. (27107)** | Bnai Zion Medical Center EC  47 Eliyahu Golomb Street.  Haifa,  31048  Israel |
| **Yeganeh, Shay, Dr. (27104)** | Baruch Padeh Poriya Medical Center EC  M.p. Lower Galilee  Tiberias,  15208  Israel  Bnai Zion Medical Center EC  47 Eliyahu Golomb Street.  Haifa,  31048  Israel  Rabin Medical Center Local EC  39 Jabotinsky St.  Petach Tikva, 49100  Israel |
| **Antonioli, Elisabetta (28111)**  **Bosi, Alberto (Former PI)** | Comitato Etico MI Area 3 - ASST Grande Ospedale Metropolitano Niguarda  PIAZZA DELL'OSPEDALE MAGGIORE 3  Milano  Lombardia, 20162  Italy  Comitato Etico Regione Toscana - Area Vasta Centro  Largo Brambilla, 3  Firenze  50134  Italy |
| **Bringhen, Sara (28101)**  **Palumbo, Antonio (Former PI)** | Comitato Etico Interaziendale A.O.U. Città della Salute e della Scienza di Torino  Corso Bramante, 88/90  Torino  Piemonte, 10126  Italy  Comitato Etico MI Area 3 - ASST Grande Ospedale Metropolitano Niguarda  PIAZZA DELL'OSPEDALE MAGGIORE 3  Milano  Lombardia, 20162  Italy |
| **Cafro, Anna Maria (28105)** | Comitato Etico MI Area 3 - ASST Grande Ospedale Metropolitano Niguarda  PIAZZA DELL'OSPEDALE MAGGIORE 3  Milano  Lombardia, 20162  Italy |
| **Cavo, Michele (28110)** | Comitato Etico Area Vasta Emilia Nord  Via Gramsci, 14  Parma  Emilia-Romagna, 43126  Italy  Comitato Etico di Area Vasta Emilia Centro della Regione Emilia-Romagna (CE-AVEC)_1  Via Albertoni, 15  Bologna  Emilia-Romagna, 40138  Italy  Comitato Etico di Area Vasta Emilia Centro della Regione Emilia-Romagna (CE-AVEC)_2  Largo Bartolo Nigrisoli, 2  Bologna  Emilia-Romagna, 40100  Italy  Comitato Etico MI Area 3 - ASST Grande Ospedale Metropolitano Niguarda  PIAZZA DELL'OSPEDALE MAGGIORE 3  Milano  Lombardia, 20162  Italy |
| **Cellini, Claudia (28102)** | Comitato Etico della Romagna - C.E.R.O.M.  VIA PIERO MARONCELLI 40  Meldola  Emilia-Romagna, 47014  Italy  Comitato Etico MI Area 3 - ASST Grande Ospedale Metropolitano Niguarda  PIAZZA DELL'OSPEDALE MAGGIORE 3  Milano  Lombardia, 20162  Italy |
| **Crugnola, Monica, MD (28112)**  **Giuliani, Nicola, Prof. (Former PI)** | Comitato Etico Area Vasta Emilia Nord  Via Gramsci, 14  Parma  Emilia-Romagna, 43126  Italy  Comitato Etico dell’Area Vasta Emilia Nord  Via Largo del Pozzo 71  Modena  Emilia-Romagna, 41124  Italy  Comitato Etico MI Area 3 - ASST Grande Ospedale Metropolitano Niguarda  PIAZZA DELL'OSPEDALE MAGGIORE 3  Milano  Lombardia, 20162  Italy  Comitato Etico Unico per la Provincia di Parma  VIA GRAMSCI 14  Parma  Emilia-Romagna, 43010  Italy |
| **Di Raimondo, Francesco (28113)** | Comitato Etico Catania 1  VIA SANTA SOFIA 78  Catania  Sicilia, 95123  Italy  Comitato Etico MI Area 3 - ASST Grande Ospedale Metropolitano Niguarda  PIAZZA DELL'OSPEDALE MAGGIORE 3  Milano  Lombardia, 20162  Italy |
| **Falcone, Antonietta Pia (28106)**  **Cascavilla, Nicola (Former PI)** | Comitato Etico dell’IRCCS Istituto Tumori “G. Paolo II”  VIALE ORAZIO FLACCO 65  Bari  Puglia, 70124  Italy  Comitato Etico MI Area 3 - ASST Grande Ospedale Metropolitano Niguarda  PIAZZA DELL'OSPEDALE MAGGIORE 3  Milano  Lombardia, 20162  Italy  Sezione del Comitato Etico IRCCS Ist. Tumori - Giovanni Paolo II di Bari c/o Fondazione Casa Solliev  Viale Cappuccini, 1  San Giovanni Rotondo  Puglia, 71013  Italy |
| **Ferrara, Felicetto (28104)** | Comitato Etico Cardarelli-Santobono  VIA ANTONIO CARDARELLI 9  Napoli  Campania, 80131  Italy  Comitato Etico MI Area 3 - ASST Grande Ospedale Metropolitano Niguarda  PIAZZA DELL'OSPEDALE MAGGIORE 3  Milano  Lombardia, 20162  Italy  Comitato Etico Università Federico II- A.O.R.N. Cardarelli  Via Sergio Pansini, 5  Napoli  Campania, 80131  Italy |
| **Galimberti, Sara (28103)**  **Petrini, Mario (Former PI)** | Comitato Etico MI Area 3 - ASST Grande Ospedale Metropolitano Niguarda  PIAZZA DELL'OSPEDALE MAGGIORE 3  Milano  Lombardia, 20162  Italy  Comitato Etico Regionale Toscana – Area Vasta Nord Ovest  VIA ROMA 67  Pisa  Toscana, 56126  Italy |
| **Lemoli, Roberto Massimo (28109)**  **Gobbi, Marco (Former PI)** | Comitato Etico MI Area 3 - ASST Grande Ospedale Metropolitano Niguarda  PIAZZA DELL'OSPEDALE MAGGIORE 3  Milano  Lombardia, 20162  Italy  Comitato Etico Regionale della Liguria  LARGO ROSANNA BENZI 10  Genova  Liguria, 16132  Italy |
| **Liberati, Anna Maria (28107)** | CER Umbria - Comitato Etico Regionale dell’Umbria  PIAZZA MENGHINI  Perugia  Umbria, 06156  Italy  Comitato Etico delle Aziende Sanitarie dell’Umbria  Via della Rivoluzione, 16  Perugia  Umbria, 06070  Italy  Comitato Etico MI Area 3 - ASST Grande Ospedale Metropolitano Niguarda  PIAZZA DELL'OSPEDALE MAGGIORE 3  Milano  Lombardia, 20162  Italy |
| **Offidani, Massimo (28114)**  **Cellini, Claudia (Former PI)**  **Ferrara, Felicetto (Former PI)** | Comitato Etico MI Area 3 - ASST Grande Ospedale Metropolitano Niguarda  PIAZZA DELL'OSPEDALE MAGGIORE 3  Milano  Lombardia, 20162  Italy  Comitato Etico Regionale delle Marche  Via Caduti del Lavoro, 40  Ancona  Marche, 60131  Italy |
| **Tucci, Alessandra (28115)**  **Rossi, Giuseppe (Former PI)** | Comitato etico di Brescia  PIAZZALE SPEDALI CIVILI 1  Brescia  Lombardia, 25123  Italy  Comitato Etico MI Area 3 - ASST Grande Ospedale Metropolitano Niguarda  PIAZZA DELL'OSPEDALE MAGGIORE 3  Milano  Lombardia, 20162  Italy |
| **Hanamoto, Hitoshi (63108)**  **Yagi, Hideo (Former PI)** | Kindai University Hospital Institutional Review Board  377-2 Onohigashi  Osakasayama  Ôsaka, 589-8511  Japan  Nara Hospital Kinki University Faculty of Medicine IRB  1248-1, Otodacho  Ikoma-City  Nara, 630-0293  Japan  National Hospital Organization Kinki, Chuo Chest Medical Center Institutional Review Board  1180, Nagasonecho, Kita-ku  Sakai-shi  591-8555  Japan  National Hospital Organization Nara Medical Center Institutional Review Board  2-789 Shichijo  Nara, Nara  630-8053  Japan |
| **Iida, Shinsuke (63106)** | Nagoya City University Hospital IRB  1-Kawasumi, Mizuho-cho, Mizuho-ku  Nagoya-City  467-8602  Japan  Nagoya Ekisaikai Hospital Institutional Review Board  4-66, Shonencho, Nakagawa-ku  Nagoya-shi  454-8502  Japan  Nagoya University Hospital Institutional Review Board  65 Tsurumai-cho, Showa-ku, Aichi  Nagoya  Aiti, 4668560  Japan |
| **Ikeda, Takashi (63109)** | Juntendo University Hospital IRB  3-1-3 Hongo  Bunkyo  Tokyo, 113-8431  Japan  Shizuoka Cancer Center organization clinical trial ethic committee  1007 Shimonagakubo, Nagaizumi-cho  Sunto-gun  Sizuoka, 411-8777  Japan |
| **Ishikawa, Takayuki (63104)** | Kobe City Medical Center General Hospital IRB  2-1-1, Minatojima-minamimachi, Chuo-ku  Kobe-City  Hyôgo, 650-0047  Japan  Kobe Red Cross Hospital Institutional Review Board  1-3-1 Wakinohama-Kaigandori, Chuo-ku  Kobe, 651-0073  Japan |
| **Iwasaki, Hiromi (63114)**  **Okamura, Seiichi (Former PI)**  **Takase, Ken (Former PI)** | National Hospital Organization Kyushu Cancer Center Institutional Review Board  3-23-1 Shiobaru  Fukuoka  Hukuoka, 815-8588  Japan  National Hospital Organization Kyushu Medical Center Institutional Review Board  1-8-1 Jigyohama, Chuo-ku  Fukuoka, 810-8563  Japan |
| **Komeno, Takuya (63110)** | National Hospital Organization Mito Medical Center Institutional Review Board  280, Sakuranosato, Ibarakimachi  Higashiibaraki-gun  311-3193  Japan |
| **Kosugi, Hiroshi (63119)** | Ogaki Municipal Hospital Institutional Review Board  4-86, Minaminokawacho  Ogaki  Gihu, 503-8502  Japan |
| **Nagafuji, Koji (63117)** | Hitachi General Hospital IRB  1-1-2 Jonancho  Hitachi  Ibaraki, 317-0077  Japan  Kurume University Hospital IRB  67 Asahi-cho  Kurume-Shi  Hukuoka  Japan |
| **Sasaki, Makoto (63115)** | Juntendo University Hospital IRB  3-1-3 Hongo  Bunkyo  Tokyo, 113-8431  Japan  Juntendo University Shizuoka Hospital IRB  1129 Nagaoka  Izunokuni-Shi  Sizuoka, 410-2211  Japan |
| **Sekiguchi, Naohiro (63107)**  **Takezako, Naoki (Former PI)** | National Hospital Organization Disaster Medical Center IRB  3256 Modori-cho, Tokyo  Tachikawa-shi  1900014  Japan |
| **Shinagawa, Atsushi (63113)** | Hitachi General Hospital IRB  1-1-2 Jonancho  Hitachi  Ibaraki, 317-0077  Japan |
| **Tsukada, Nobuhiro (63101)**  **Suzuki, Kenshi, MD (Former PI)** | Japanese Red Cross Medical Center IRB  4-1-22 Hiroo  Shibuya-ku  Tokyo, 150-8935  Japan |
| **Yamamura, Ryosuke (63112)**  **Ohta, Kensuke (Former PI)** | Nakatsu Hospital Institutional Review Board  2-10-39 Shibata, Kita-ku  Osaka  530-0012  Japan |
| **Eom, HyeonSeok (29106)** | National Cancer Center IRB  323 Ilsan-ro Ilsandong-gu  Goyang-si  Gyeonggido, 10408  Korea, Republic of |
| **Kim, Jin Seok (29105)** | Severance Hospital, Yonsei University Health System IRB  Yeonsedaehakgyo  Seodaemun-Gu  Seoul Teugbyeolsi, 03722  Korea, Republic of |
| **Kim, KiHyun (29104)** | Samsung Medical Center IRB  81 Irwon-dong Gangnam-gu  Seoul, 06351  Korea, Republic of |
| **Lee, Jae Hoon (29103)** | Gachon University Gil Medical Center IRB  Gacheondaehakgyo Gilbyeongweon  Namdong-Gu  Incheon Gwang'yeogsi, 21565  Korea, Republic of |
| **Min, Chang Ki (29101)** | The Catholic University of Korea, Seoul St. Mary's Hospital IRB 222 Banpo daero Seocho gu  Seoul, 06591  Korea, Republic of  The Catholic University of Korea, St. Vincent's Hospital IRB  St. Vincent Hospital  Suwon, 442-723  Korea, Republic of |
| **Yoon, Sung-Soo (29102)** | Seoul National University Hospital IRB  101 Daehak-Ro  Jongno-Gu  Seoul Teugbyeolsi, 03080  Korea, Republic of |
| **Flores Jimenez, Juan Antonio, MD (35101)** | Comite de Etica en Investigación del Centro de Investigacion Farmaceutica Especializada de Occidente  Av. Vallarta No. 1670  Guadalajara  Guadalajara  Jalisco, 44160  Mexico  Comité de Ética en Investigación del Hospital Hispano S.A. de C.V.  Pedro Moreno Numero 934  Guadalajara  Jalisco, 44100  Mexico  Comité de Ética en Investigación de Unidad Clínica de Bioequivalencia S. de R.L. de C.V.  Av. Alemania No. 1361  Guadalajara  Jalisco, 44190  Mexico |
| **Gómez Almaguer, David, MD (35102)** | Comité de Ética en Investigación de la Facultad de Medicina de la UANL y Hospital Universitario "Dr. Jose Eleuterio Gonzalez"  Av. Francisco I. Madero y Gonzalitos S/N, Colonia Mitras Centro  Monterrey  Nuevo León, 64460  Mexico |
| **Martinez Baños, Deborah Maria, MD (35105)** | Comite de Etica en Investigacion del Instituto Nacional de Ciencias Medicas y Nutricion  Vasco de Quiroga 15  Mexico City  14000  Mexico  Comité de Ética Investigación Instituto Nacional de Ciencias Medicas y Nutricion Salvador Zubiran  Vasco de Quiroga 15  Ciudad de Mexico  14080  Mexico |
| **Ramirez Romero, Eva Fabiola, MD (35104)** | Comité de Ética en Investigación Oaxaca Site Management Organization, S.C.  Humboldt 302 Col. Centro  Oaxaca, Oaxaca  68000  Mexico |
| **Czyz, Jaroslaw, MD, PhD (42105)** | Komisja Bioetyczna Slaskiej Izby Lekarskiej w Katowicach  Grazynskiego 49  Katowice  Slaskie, 40-126  Poland |
| **Grosicki, Sebastian, MD, PhD (42102)** | Komisja Bioetyczna Slaskiej Izby Lekarskiej w Katowicach  Grazynskiego 49  Katowice  Slaskie, 40-126  Poland |
| **Jedrzejczak, Wieslaw, Prof., MD, PhD (42106)** | Komisja Bioetyczna Slaskiej Izby Lekarskiej w Katowicach  Grazynskiego 49  Katowice  Slaskie, 40-126  Poland |
| **Usnarska-Zubkiewicz, Lidia, Prof., MD, PhD (42103)** | Komisja Bioetyczna Slaskiej Izby Lekarskiej w Katowicach  Grazynskiego 49  Katowice  Slaskie, 40-126  Poland |
| **Bernardo, Maria Manuela Henriques (43105)** | Comissão de Ética para a Investigação Clínica – CEIC  Avenida do Brasil, 53  Lisboa  Lisboa, 1749-004  Portugal |
| **Domingues, Nelson (43103)** | Comissão de Ética para a Investigação Clínica – CEIC  Avenida do Brasil, 53  Lisboa  Lisboa, 1749-004  Portugal |
| **Marques, Herlander Jose de Resende**  **(43102)** | Comissão de Ética para a Investigação Clínica – CEIC  Avenida do Brasil, 53  Lisboa  Lisboa, 1749-004  Portugal |
| **Neves, Manuel, MD (43107)**  **Pires João, Cristina Maria Godinho**  **(Former PI)** | Comissão de Ética para a Investigação Clínica – CEIC  Avenida do Brasil, 53  Lisboa  Lisboa, 1749-004  Portugal |
| **Pereira Gonçalves, Cristina Maria Andrade (43101)** | Comissão de Ética para a Investigação Clínica – CEIC  Avenida do Brasil, 53  Lisboa  Lisboa, 1749-004  Portugal |
| **Trigo, Fernanda (43104)** | Comissão de Ética para a Investigação Clínica – CEIC  Avenida do Brasil, 53  Lisboa  Lisboa, 1749-004  Portugal |
| **Vargas, Fernanda, MD (43106)**  **Trindade, Maria do Céu (Former PI) Marques da Costa, Ricardo Jorge Seitil (Former PI)** | Comissão de Ética para a Investigação Clínica – CEIC  Avenida do Brasil, 53  Lisboa  Lisboa, 1749-004  Portugal |
| **Luchinin, Alexander, MD (46105)**  **Sherman, Nailya, MD (Former PI)** | Ethics Committee at Kirov Research Institute of Haematology and Blood Transfusion  Krasnoarmeyskaya Ulitsa 72  Kirov  610027  Russian Federation  Ethics Committee at State Medical and Preventive Treatment Institution Kirov Regional Clinical Oncol  Prospect Stroiteley, 23  Kirov  610021  Russian Federation  The RF MoH, Department of State Regulation of Circulation of Medicines, Ethics Council  Rakhmanovsky Pereulok 3  Moscow  127994  Russian Federation |
| **Medvedeva, Nadezhda, MD, PhD (46106)** | Ethics Committee at City Clinical Hospital #31  Prospect Dinamo, 3  St. Petersburg  197110  Russian Federation  The RF MoH, Department of State Regulation of Circulation of Medicines, Ethics Council  Rakhmanovsky Pereulok 3  Moscow  127994  Russian Federation |
| **Pristupa, Alexander, MD, PhD (46101)** | Ethics Committee at Ryazan Regional Clinical Hospital  Ulitsa Internatsionalnaya, 3a  Ryazan  390039  Russian Federation  The RF MoH, Department of State Regulation of Circulation of Medicines, Ethics Council  Rakhmanovsky Pereulok 3  Moscow  127994  Russian Federation |
| **Shmidt, Alexander (46102)**  **Abdulkadyrov, Kudrat, Prof., Dr. Med. Sci., PhD, MD (Former PI)** | EC at Russian Research Institute of Haematology and Transfusiology of Medicobiologic Agency  Ulitsa Vtoraya Sovetskaya, 16  St. Petersburg  193024  Russian Federation  The RF MoH, Department of State Regulation of Circulation of Medicines, Ethics Council  Rakhmanovsky Pereulok 3  Moscow  127994  Russian Federation |
| **Vladimirov, Vladimir, MD, PhD, Dr. Med. Sci. (46104)** | Ethics Committee at Stavropol Regional Clinical Oncology Centre Pyatigorsk Affiliate  Ulitsa Kalinina, 31  Pyatigorsk  357502  Russian Federation  The RF MoH, Department of State Regulation of Circulation of Medicines, Ethics Council  Rakhmanovsky Pereulok 3  Moscow  127994  Russian Federation |
| **Bila, Jelena, Prof., Dr. (47102)** | Ethics Committee of Serbia  Vojvode Stepe 458  Belgrade, Belgrade  11152  Serbia  LEC University Clinical Centre Serbia  Pasterova 2  Belgrade, Belgrade  11000  Serbia |
| **Cojbasic, Irina (47101)**  **Macukanovic-Golubovic, Lana (Former PI)** | Ethics Committee of Serbia  Vojvode Stepe 458  Belgrade, Belgrade  11152  Serbia  LEC University Clinical Centre Nis  Bulevar Zorana Djindjica 48  Niš  18000  Serbia |
| **Djurdjevic, Predrag, Dr. (47103)** | Ethics Committee of Serbia  Vojvode Stepe 458  Belgrade, Belgrade  11152  Serbia  LEC Clinical Centre Kragujevac  Zmaj Jovina 30  Kragujevac  Šumadijski okrug, 34000  Serbia |
| **Markovic, Olivera, Dr. (47104)** | Ethics Committee of Serbia  Vojvode Stepe 458  Belgrade, Belgrade  11152  Serbia  LEC Clinical Hospital Centre Bezanijska Kosa  Bezanijska Kosa bb  Belgrade, Belgrade  11070  Serbia |
| **Chng, Wee Joo (48101)** | Domain Specific Review Board  Nexus@One-North (South Tower)  Singapore  138543  Singapore |
| **Nagarajan, Chandramouli (48102)**  **Yunxin, Chen (Former PI)**  **Gopalakrishnan, Sathish Kumar (Former PI)** | Domain Specific Review Board  Nexus@One-North (South Tower)  Singapore  138543  Singapore |
| **Cohen, Graham Lawrence, Dr. (50103)** | Pharma-Ethics Research Ethics Committee  123 Amcor Road  Pretoria  Gauteng, 0157  South Africa |
| **McDonald, Andrew Bruce, Dr. (50101)** | Pharma-Ethics Research Ethics Committee  123 Amcor Road  Pretoria  Gauteng, 0157  South Africa |
| **Rapoport, Bernardo Leon, Dr. (50104)** | Pharma-Ethics Research Ethics Committee  123 Amcor Road  Pretoria  Gauteng, 0157  South Africa |
| **Amor, Adrian Alegre (51110)** | CEIC Hospital Universitario de la Princesa  Calle Diego de León, 62  Madrid, Madrid  28006  Spain  CEIC Hospital Universitario Germans Trias i Pujol  Carretera Canyet S  Badalona  Barcelona, 08916  Spain |
| **Arnao, Mario (51111)**  **Ramos, Isidro Jarque (Former PI)** | CEIC Hospital Universitario Germans Trias i Pujol  Carretera Canyet S  Badalona  Barcelona, 08916  Spain  CEIC Hospital Universitario y Politecnico la Fe  Avenida Fernado Abril Martorell, 106  Valencia, Valencia  46026  Spain |
| **Blade Creixenti, Joan (51112)** | CEIC Hospital Universitario Germans Trias i Pujol  Carretera Canyet S  Badalona  Barcelona, 08916  Spain  CEIm Hospital Clinic de Barcelona  Calle Villarroel, 170  Barcelona, Barcelona  08036  Spain |
| **De Arriba de la Fuente, Felipe (51104)** | CEIC Hospital Universitario Germans Trias i Pujol  Carretera Canyet S  Badalona  Barcelona, 08916  Spain  CEIC Hospital General Universitario Morales Meseguer  Avenida Marques de los Velez s/n  Murcia, Murcia  30008  Spain |
| **Encinas Rodriguez, Cristina (51108)** | CEIC Hospital Universitario Germans Trias i Pujol  Carretera Canyet S  Badalona  Barcelona, 08916  Spain  CEIC Hospital General Universitario Gregorio Marañon  Calle Doctor Esquerdo, 46  Madrid, Madrid  28007  Spain |
| **Granell Gorrochateguí, Miquel (51103)** | CEIC Hospital Santa Creu i Sant Pau  Avenida Sant Antoni Maria Claret, 167  Barcelona, Barcelona  08025  Spain  CEIC Hospital Universitario Germans Trias i Pujol  Carretera Canyet S  Badalona  Barcelona, 08916  Spain |
| **Hernandez Rivas, Jose Angel (51109)** | CEIC Hospital General Universitario Gregorio Marañon  Calle Doctor Esquerdo, 46  Madrid, Madrid  28007  Spain  CEIC Hospital Universitario Germans Trias i Pujol  Carretera Canyet S  Badalona  Barcelona, 08916  Spain |
| **Lopez de la Guia, Ana (51101)** | CEIC Hospital Universitario Germans Trias i Pujol  Carretera Canyet S  Badalona  Barcelona, 08916  Spain  CEIC Hospital Universitario La Paz  Paseo de la Castellana, 261  Madrid, Madrid  28046  Spain |
| **Martinez Chamorro, Carmen (51113)** | CEIC Hospital Puerta de Hierro Majadahonda  C/ Joaquín Rodrigo, 2  Majadahonda, Madrid  28222  Spain  CEIC Hospital Universitario Germans Trias i Pujol  Carretera Canyet S  Badalona  Barcelona, 08916  Spain |
| **Mateos Manteca, Maria Victoria (51102)** | CEIC Área de Salud de Salamanca  Paseo De San Vicente 58-182  Salamanca  Castilla y León, 37007  Spain  CEIC Hospital Universitario Germans Trias i Pujol  Carretera Canyet S  Badalona  Barcelona, 08916  Spain |
| **Oriol Rocafiguera, Albert, Dr. (51105)** | CEIC Hospital Universitario Germans Trias i Pujol  Carretera Canyet S  Badalona  Barcelona, 08916  Spain |
| **Perez de Oteyza, Jaime (51106)** | CEIC Grupo Hospital de Madrid  Avenida Monteprincipe, 25  Boadilla del Monte  Madrid, 28660  Spain  CEIC Hospital Universitario Germans Trias i Pujol  Carretera Canyet S  Badalona  Barcelona, 08916  Spain |
| **Rodriguez Otero, Paula (51107)** | CEIC de Navarra  Calle Irunlarrea, 3  Pamplona  Navarra, 31008  Spain  CEIC Hospital Universitario Germans Trias i Pujol  Carretera Canyet S  Badalona  Barcelona, 08916  Spain |
| **Hansson, Markus (52102)** | Etikprövningsmyndigheten  von Kraemers allé 4  Uppsala  Uppsala lan  SE-752 37  Sweden  Regionala etikprövningsnämnden i Stockholm  Tomtebodavägen 18A, plan 3  Solna  SE-17165  Sweden |
| **Nahi, Hareth (52101)** | Etikprövningsmyndigheten  von Kraemers allé 4  Uppsala  Uppsala lan  SE-752 37  Sweden  Regionala etikprövningsnämnden i Stockholm  Tomtebodavägen 18A, plan 3  Solna  SE-17165  Sweden |
| **Stromberg, Olga (52104)** | Regionala etikprövningsnämnden i Stockholm  Tomtebodavägen 18A, plan 3  Solna  SE-17165  Sweden |
| **Rauch, Daniel, Dr. med. (53101)** | Kantonale Ethikkommission Bern (KEK)  Murtenstraße 31  Bern  3010  Switzerland |
| **Hsiao, Hui-Hua (54103)**  **Liu, Ta-Chih (Former PI)** | Institutional Review Board Kaohsiung Medical University Chung-Ho Memorial Hospital  No.100, Tzyou 1st Road  Kaohsiung City  807  Taiwan, Province of China |
| **Huang, Shang-Yi (54102)** | Institution Review Board of National Taiwan University Hospital  No.1, Changde-de Street, Zhongzheng Dist  Taipei  100  Taiwan, Province of China  National Taiwan University Hospital (NTUH) Research Ethics Committee (REC)  No. 33 Linsen South Road  Taipei City  100  Taiwan, Province of China  Research Ethics Committee of National Taiwan University Hospital  No.7 Chung-Shan South Road  Taipei  Taipei, 10002  Taiwan, Province of China |
| **Wen-Li, Hwang (54101)** | Institutional Review Board of Taichung Veterans General Hospital  No. 1650 Taiwan Boulevard, Sec. 4  Taichung  40705  Taiwan, Province of China |
| **Chuncharunee, Suporn, MD (64101)** | The Ethical Clearance Committee on Human Rights Related to Researchers Involving Human Subjects, Faculty of Medicine  270 Rama Vi Road  Bangkok  Krung Thep Maha Nakhon, 10400  Thailand  Ramathibodi Hospital Ethics Committee  Rama Vi Road, Faculty Of Medicine Ramathibodi Hospital  Bangkok  Krung Thep Maha Nakhon, 10400  Thailand |
| **Lawasut, Panisinee, MD (64103)**  **Na Nakorn, Thanyaphong, MD (Former PI)** | The Ethical Clearance Committee on Human Rights Related to Researchers Involving Human Subjects, Faculty of Medicine  270 Rama Vi Road  Bangkok  Krung Thep Maha Nakhon, 10400  Thailand  The Institutional Review Board of the Faculty of Medicine, Chulalongkorn University  1873 Rama 4 Road  Bangkok  Krung Thep Maha Nakhon, 10330  Thailand |
| **Norasetthada, Lalita, MD (64102)** | Research Ethics Committee, Faculty of Medicine, Chiang Mai University  110 Intavaroros Road  Chiang Mai, Chiang Mai  50200  Thailand |
| **Beksac, Meral, Prof., Dr. (55101)** | Ankara University Medical Faculty Ethics Committee  Sihhiye  Ankara, Ankara  06100  Turkey |
| **Besisik, Sevgi Kalayoglu (55104)** | Ankara University Medical Faculty Ethics Committee  Sihhiye  Ankara, Ankara  06100  Turkey |
| **Ozsan, Guner Hayri (55103)** | Ankara University Medical Faculty Ethics Committee  Sihhiye  Ankara, Ankara  06100  Turkey |
| **Ayto, Robert Michael, Dr. (57128)** | North East – York Research Ethics Committee  NHSBT Newcastle Blood Donor Centre  Holland Drive  Newcastle Upon Tyne, NE2 4NQ  United Kingdom |
| **Basu, Supratik, Dr. (57116)** | North East – York Research Ethics Committee  NHSBT Newcastle Blood Donor Centre  Holland Drive  Newcastle Upon Tyne, NE2 4NQ  United Kingdom |
| **Benjamin, Reuben, Dr. (57118)** | North East – York Research Ethics Committee  NHSBT Newcastle Blood Donor Centre  Holland Drive  Newcastle Upon Tyne, NE2 4NQ  United Kingdom |
| **Bird, Jennifer M., Dr. (57115)** | North East – York Research Ethics Committee  NHSBT Newcastle Blood Donor Centre  Holland Drive  Newcastle Upon Tyne, NE2 4NQ  United Kingdom United Kingdom |
| **Boyd, Kevin, Dr. (57105)**  **Kaiser, Martin, Dr. (Former PI)** | North East – York Research Ethics Committee  NHSBT Newcastle Blood Donor Centre  Holland Drive  Newcastle Upon Tyne, NE2 4NQ  United Kingdom |
| **Brown, Rachel, Dr. (57120)**  **Rocci, Alberto, Dr. (Former PI)** | North East – York Research Ethics Committee  NHSBT Newcastle Blood Donor Centre  Holland Drive  Newcastle Upon Tyne, NE2 4NQ  United Kingdom |
| **Bygrave, Ceri Ann, Dr. (57108)** | North East – York Research Ethics Committee  NHSBT Newcastle Blood Donor Centre  Holland Drive  Newcastle Upon Tyne, NE2 4NQ  United Kingdom |
| **Chaidos, Aristeidis, Dr. (57101)** | North East – York Research Ethics Committee  NHSBT Newcastle Blood Donor Centre  Holland Drive  Newcastle Upon Tyne, NE2 4NQ  United Kingdom |
| **El-Agnaf, Moulod Ramadan, Dr. (57126)** | North East – York Research Ethics Committee  NHSBT Newcastle Blood Donor Centre  Holland Drive  Newcastle Upon Tyne, NE2 4NQ  United Kingdom |
| **Floro, Lajos, Dr. (57109)**  **Kagdi, Husseini, Dr. (Former PI)**  **Zakout, Ghada Ahmed, Dr. (Former PI)**  **Ayto, Robert Michael, Dr. (Former PI)** | North East – York Research Ethics Committee  NHSBT Newcastle Blood Donor Centre  Holland Drive  Newcastle Upon Tyne, NE2 4NQ  United Kingdom |
| **Gabriel, Ian Howell, Dr. (57124)** | North East – York Research Ethics Committee  NHSBT Newcastle Blood Donor Centre  Holland Drive  Newcastle Upon Tyne, NE2 4NQ  United Kingdom United Kingdom |
| **Garg, Mamta, Dr. (57106)** | North East – York Research Ethics Committee  NHSBT Newcastle Blood Donor Centre  Holland Drive  Newcastle Upon Tyne, NE2 4NQ  United Kingdom |
| **Hall, Rachel Louise (57122)** | North East – York Research Ethics Committee  NHSBT Newcastle Blood Donor Centre  Holland Drive  Newcastle Upon Tyne, NE2 4NQ  United Kingdom |
| **Jabbar Al-Obaidi, Magda, Dr (57123)** | North East – York Research Ethics Committee  NHSBT Newcastle Blood Donor Centre  Holland Drive  Newcastle Upon Tyne, NE2 4NQ  United Kingdom |
| **Moore, Sally, Dr. (57104)**  **Crowe, Josephine, Dr. (Former PI)** | North East – York Research Ethics Committee  NHSBT Newcastle Blood Donor Centre  Holland Drive  Newcastle Upon Tyne, NE2 4NQ  United Kingdom |
| **Kaczmarski, Richard Stanislaw, Dr. (57114)** | North East – York Research Ethics Committee  NHSBT Newcastle Blood Donor Centre  Holland Drive  Newcastle Upon Tyne, NE2 4NQ  United Kingdom |
| **Kishore, Bhuvan, Dr. (57119)** | North East – York Research Ethics Committee  NHSBT Newcastle Blood Donor Centre  Holland Drive  Newcastle Upon Tyne, NE2 4NQ  United Kingdom |
| **Kothari, Jaimal Jaysukhlal, Dr. (57110)** | North East – York Research Ethics Committee  NHSBT Newcastle Blood Donor Centre  Holland Drive  Newcastle Upon Tyne, NE2 4NQ  United Kingdom United Kingdom |
| **Lindsay, Jindriska (57111)** | North East – York Research Ethics Committee  NHSBT Newcastle Blood Donor Centre  Holland Drive  Newcastle Upon Tyne, NE2 4NQ  United Kingdom |
| **Oakervee, Heather, Dr. (57102)** | North East – York Research Ethics Committee  NHSBT Newcastle Blood Donor Centre  Holland Drive  Newcastle Upon Tyne, NE2 4NQ  United Kingdom |
| **Sati, Hamdi Idres Abdulla, Dr. (57103)** | North East – York Research Ethics Committee  NHSBT Newcastle Blood Donor Centre  Holland Drive  Newcastle Upon Tyne, NE2 4NQ  United Kingdom |
| **Whiteway, Alastair John, Dr. (57117)** | North East – York Research Ethics Committee  NHSBT Newcastle Blood Donor Centre  Holland Drive  Newcastle Upon Tyne, NE2 4NQ  United Kingdom |
| **Zhelyazkova, Antonina Gancheva, Dr. (57107)**  **Osborne, David, Dr. (Former PI)** | North East – York Research Ethics Committee  NHSBT Newcastle Blood Donor Centre  Holland Drive  Newcastle Upon Tyne, NE2 4NQ  United Kingdom |
| **Bailey, Samuel, MD (58119)**  **Ghazal, Hassan (Former PI)** | Quorum Review IRB  1501 Fourth Ave, Suite 800  Seattle, WA 98101  United States |
| **Boccia, Ralph V., MD (58105)** | Advarra IRB  6940 Columbia Gateway Dr.,  Ste 110  Columbia, Maryland  21046-2878  United States  Quorum Review IRB  1501 Fourth Ave, Suite 800  Seattle, WA 98101  United States |
| **Chay, Christopher, MD (58102)** | Mission Health Cancer Institutional Review Board  509 Biltmore Ave.  Asheville, NC 28801  United States |
| **Coleman, Morton, MD (58115)** | Quorum Review IRB  1501 Fourth Ave, Suite 800  Seattle, WA 98101  United States |
| **Comenzo, Raymond, MD (58123)** | Western Institutional Review Board  1019 39th Ave SE, Suite 120  Puyallup, WA 98374  United States |
| **Ibrahim, Emad, MD (58101)** | Quorum Review IRB  1501 Fourth Ave, Suite 800  Seattle, WA 98101  United States |
| **Lee, Arielle S., MD (58114)**  **Droder, Robert, MD (Former PI)** | East Texas Medical Center Regional Healthcare Systems IRB  721 Clinic Drv.  Tyler, TX 75701  United States |
| **Lonial, Sagar, MD (58112)** | Western Institutional Review Board  1019 39th Ave SE, Suite 120  Puyallup, WA 98374  United States |
| **Moss, Robert A., MD (58120)** | Quorum IRB  1501 Fourth Avenue  Seattle, Washington  98101  United States |
| **Wender, Donald B., MD (58104)** | Siouxland Institutional Review Board  Health, Inc.  230 Nebraska St. Sioux City, IA 51102  United States |
| **Yates, Todd J., DO (58117)** | Quorum Review IRB  1501 Fourth Ave, Suite 800  Seattle, WA 98101  United States |
| **Zia, Maryam, MD (58107)**  **Sumoza, Luis David (Former PI)** | Cook County Health and Hospital System Institutional Review Board  1950 W Polk St Rm 9303  Chicago, Illinois  60612-3723  United States |
